# Oxide semiconductor in a neuromorphic chromaticity communication loop for extreme environment exploration

**DOI:** 10.1126/sciadv.adu3576

**Published:** 2025-05-16

**Authors:** Shangda Qu, Qianbo Yu, Chengpeng Jiang, Taoyu Zou, Honghuan Xu, Longlong Zhang, Mengze Tao, Qingshan Zhu, Song Zhang, Cong Geng, Mingjian Yuan, Yong-Young Noh, Wentao Xu

**Affiliations:** ^1^ Institute of Photoelectronic Thin Film Devices and Technology, Key Laboratory of Photoelectronic Thin Film Devices and Technology of Tianjin, College of Electronic Information and Optical Engineering, Engineering Research Center of Thin Film Photoelectronic Technology of Ministry of Education, Smart Sensing Interdisciplinary Science Center, Nankai University, Tianjin 300350, China.; ^2^ Shenzhen Research Institute of Nankai University, Shenzhen 518000, China.; ^3^ Department of Chemical Engineering, Pohang University of Science and Technology, Pohang 37673, Republic of Korea.; ^4^ State Key Laboratory of Space Weather, National Space Science Center, Chinese Academy of Sciences, Beijing 100190, China.; ^5^ Department of Chemistry, Nankai University, Tianjin 300071, China.

## Abstract

Space exploration, particularly in the extreme space environment, has gained increasing attention. Networked robots capable of real-time environmental perception and autonomous collaboration offer a promising alternative for executing complex precision tasks. Consequently, achieving local reliable communication and preparing irradiation-tolerant materials are essential. Here, we demonstrate a cephalopod-inspired neuromorphic loop that enables chromaticity communication between individual near-sensor processing units. A programmatically aligned aluminum zinc oxide nanofiber array was fabricated and used as conductive channels that can withstand prolonged (~10^4^ seconds) and high-dose (~5 × 10^15^ ions per square centimeter) proton irradiation. The neuromorphic loop, with capabilities in environmental perception, event-driven processing, adaptive learning, and chromaticity communication, enables the self-driven collaboration of robotic hands based on tactile feedback and ensures reliable mobile links for drone flight control. This work pioneers a direction in neuromorphic visible light communication and marks important progress in the field of biomimetic intelligence.

## Teaser

A neuromorphic chromaticity communication loop demonstrates potential for extreme environment exploration.

## INTRODUCTION

Planetary exploration is a captivating and essential endeavor that advances our understanding of the solar system and explores potential resources (*1*). However, the space environment presents far greater challenges than Earth’s surface, with high-energy particles and radiation, making it inhospitable for human activities (*2*–*4*). In addition to these physical challenges, the vast distance from Earth leads to communication instability, long delays, and interference with radio frequency signals, highlighting the need for reliable, real-time auxiliary communication methods, especially for space exploration missions (*5*, *6*). Therefore, networked robots used in these missions require the capability to perceive environmental information and cooperate autonomously in real time to execute complex group tasks accurately under these extreme environments (*7*–*9*).

In nature, cephalopods, such as octopuses, cuttlefish, and squids, can transform perceived external information into surface color changes through neural processing, thereby achieving camouflage and intraspecies communication (*10*, *11*). For example, the Humboldt squid (*Dosidicus gigas*) living in the deep sea has subcutaneous luminescent organs throughout muscle tissue that can express informative body backlight patterns (*12*), facilitating rapid and efficient visible light communication within its group. This biological mechanism allows for seamless integration of perception, processing, and communication, crucial for coordinating complex group behavior in highly dynamic environments (*12*, *13*). By mimicking group communication governed by neural polymorphism, a neuromorphic system that combines perception, processing, and chromaticity-encoded communication can be designed. This system can not only achieve environmental perception, event-driven processing, and adaptive learning but also supplement radio frequency communication, enhancing the stability and reliability of mobile communication links in space exploration.

In addition, electronic systems used in space missions are often at risk of damage due to prolonged exposure to cosmic radiation, predominantly composed of protons, which can greatly reduce the life span of these missions (*14*). The operational stability of space electronic systems relies heavily on the electrical properties of the semiconductor materials (*3*, *15*). Oxide semiconductors with excellent radiation resistance are gaining attention for space applications (*16*–*18*), showing promise for neuromorphic systems and devices used in space. In particular, when oxide semiconductors are made into nanofibers, they show high sensitivity in neuromorphic devices using ion gels due to the large specific surface area. However, oxide nanofibers face challenges related to verifying their high stability in space and achieving ordered arrangement over large areas, which limit their integration into neuromorphic systems and devices for space applications.

Here, we introduce a cephalopod-inspired neuromorphic chromaticity communication loop (NCCL) capable of near-sensor processing, neuromorphic chromaticity encoding, and visible light communication for information acquisition and transmission in planetary exploration. The NCCL uses an artificial neural circuit as the transmitter and robots equipped with photodetectors as the receiver. To this end, programmatically aligned aluminum zinc oxide (AZO) nanofibers serve as synaptic transistor channels, offering resistance to prolonged (~10^4^ s) and high-dose (~5 × 10^15^ ions/cm^2^) proton irradiation. Inspired by cephalopod neural polymorphism, the artificial neural circuit achieves tactile sensation–to–optical expression conversion and chromaticity-encoded rapid communication. The NCCL is applied to enable self-driven collaboration of robotic hands based on tactile feedback and ensure reliable mobile links for drone flight control. In addition, the NCCL perceives tactile environmental information through an event-driven process, enabling adaptive learning and reliable communication between mobile objects. The NCCL offers supplementary options for radio frequency communication and robotic networking in extreme environments like space.

## RESULTS

### Concept of the NCCL

**Fig. 1.**
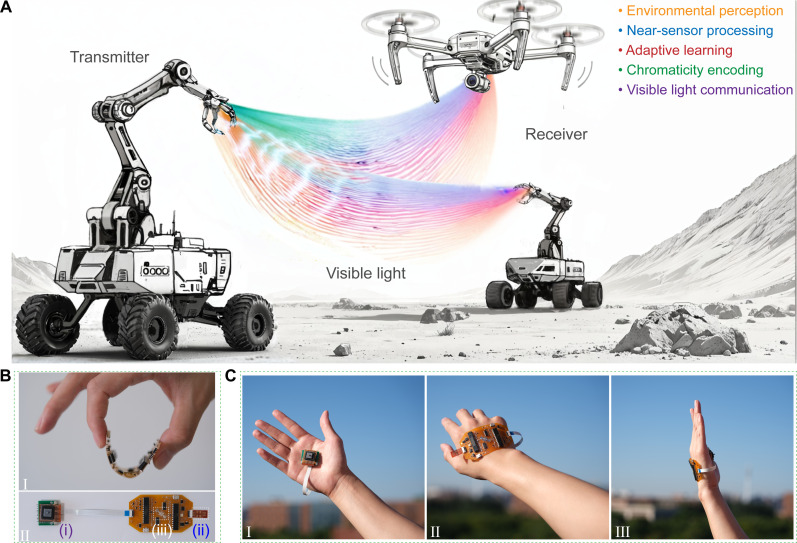
Concept of the NCCL. (**A**) Schematic of the NCCL for planetary exploration. (**B**) Digital images of the flexible artificial neural circuit. (i) Multifunctional tactile sensor. (ii) AZO nanofiber–exploiting synaptic transistor (AFST). (iii) Hybrid quantum dot (QD) light-emitting diode (QLED). (**C**) Digital images of the flexible artificial neural circuit adhered to the surfaces of the human body.

Cephalopods living in the deep ocean use bioluminescent backlighting to display various colors and communicate with each other in complex social scenarios (*12*), with their rapid neural polymorphism controlled by neural mechanisms (*13*). To mimic this efficient group communication in extreme conditions, we designed an NCCL that links a transmitter and a receiver through chromaticity-encoded visible light, implementing near-sensor processing for networked robots in planetary exploration (Fig. 1A). The artificial neural circuit serving as the transmitter has the potential for high flexibility and can be firmly attached to the surfaces of exploration robots or the human body (Fig. 1, B and C). This transmitter converts tactile sensations into optical expressions through neuromorphic chromaticity encoding, enabling diverse environmental perception, event-driven processing, and adaptive learning capabilities. The receiver, comprising photodetectors and robots (a manipulator and a drone), responds to the transmitter to execute complex tasks.

### Programmatically aligned AZO nanofibers with irradiation tolerance

**Fig. 2.**
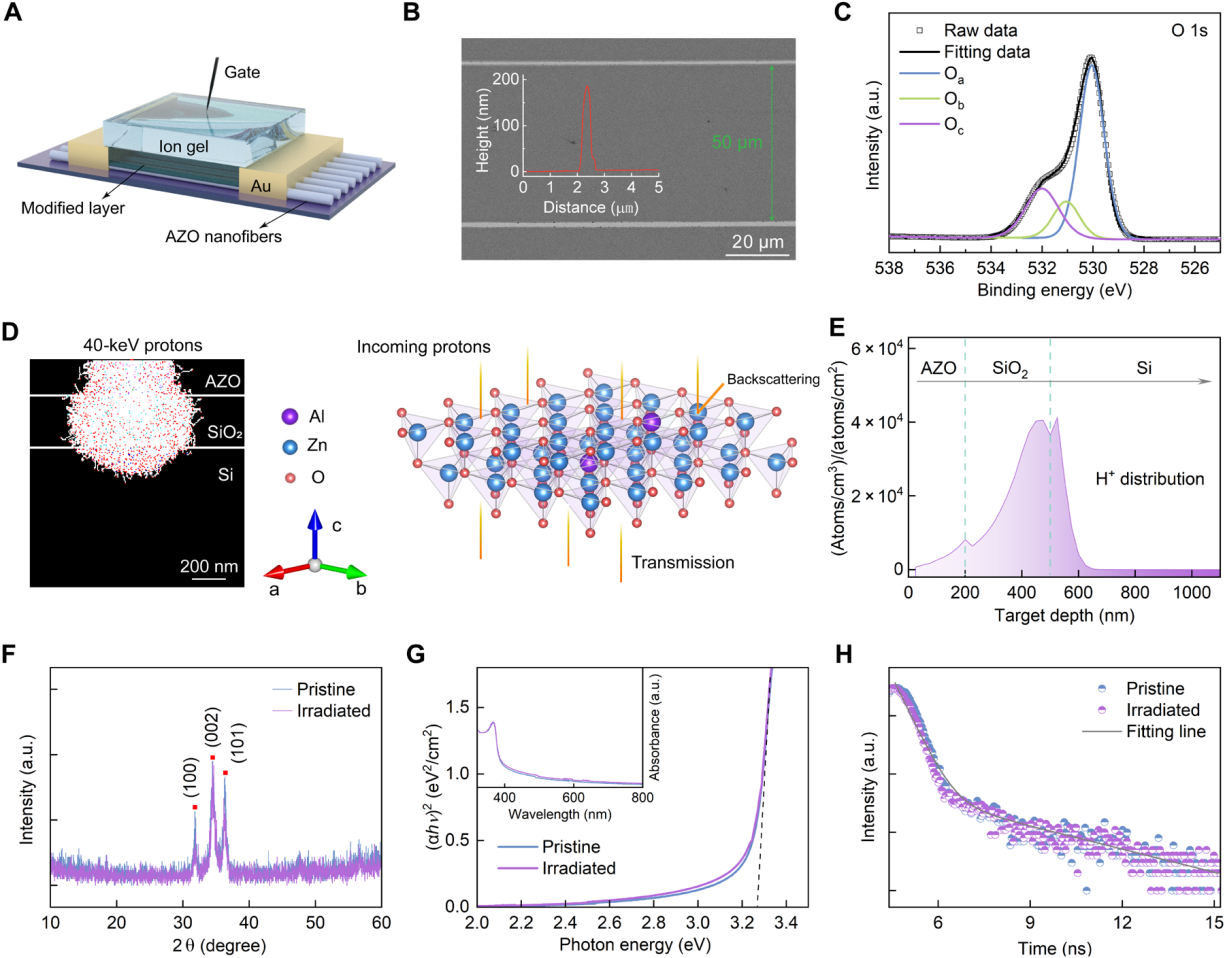
Programmatically aligned AZO nanofibers with irradiation tolerance. (**A**) Schematic of the AFST. (**B**) SEM image of AZO nanofiber array. Inset: Cross-sectional analysis of an AFM image of a single AZO nanofiber. (**C**) O 1s XPS spectrum of AZO nanofibers. (**D**) Schematic of the possible paths of proton beam irradiated onto AZO nanofibers (left) and the calculated crystal structure of AZO nanofibers (right). (**E**) SRIM-predicted incident depth profile of protons. (**F**) XRD patterns of the pristine and irradiated AZO nanofibers. (**G**) Plot of (α*hʋ*)^2^ as a function of photon energy for the pristine and irradiated AZO nanofibers. Inset: Absorbance of the pristine and irradiated AZO nanofibers. (**H**) TRPL decay of the pristine and irradiated AZO nanofibers. a.u., arbitrary units.

Highly aligned AZO nanofibers, serving as the channels in the AZO nanofiber–exploiting synaptic transistors (AFSTs), were programmatically fabricated using an electrohydrodynamic nanowire printer (Fig. 2A). Scanning electron microscopy (SEM) images show that the spacing between two adjacent highly aligned AZO nanofibers is ~50 μm (Fig. 2B), and the diameter of each nanofiber is ~600 nm (fig. S1A). Atomic force microscopy (AFM) image and corresponding cross-sectional analysis illustrate the three-dimensional (3D) shape and the height (~200 nm) of a single AZO nanofiber, respectively (fig. S1B and inset of Fig. 2B). High-resolution transmission electron microscope (TEM) images confirm the polycrystalline structure of AZO nanofibers, and high-angle annular dark-field scanning TEM images, accompanied by elemental mapping images, demonstrate the uniform distribution of Al, Zn, and O elements within AZO (fig. S2). X-ray photoelectron spectroscopy (XPS) spectra show that the binding energy of Al 2p, Zn 2p_1/2_, and Zn 2p_3/2_ peaks is centered at ~74.20, ~1044.50, and ~1021.35 eV, respectively (fig. S3). The O 1s spectrum is divided into three peaks, denoted as O_a_ (~530.05 eV), O_b_ (~531.05 eV), and O_c_ (~532.00 eV), which are related to metal-oxide bonds, oxygen vacancies, and oxygen in impurities, respectively (Fig. 2C). The O_b_ peak contributes to the mimicry of synaptic plasticity (*19*).

The structural and optical stability of AZO nanofibers before and after proton irradiation was investigated to verify their irradiation tolerance. First-principles calculations, using density functional theory within the Vienna Ab initio Simulation Package (*20*–*22*), were conducted to determine the structural and electronic properties of AZO. The calculations demonstrated that the crystal structure of AZO is hexagonal wurtzite with slight disorder, and the density of states at the Fermi level is mainly contributed by the s-electron of Al, Zn, and O (Fig. 2D and fig. S4). AZO nanofibers were irradiated with ~40-keV protons at a dose of ~5 × 10^15^ ions/cm^2^ for a duration of ~10^4^ s, which can penetrate fibers thoroughly or backscatter due to collision (Fig. 2D). A software package named Stopping and Range of Ions in Matter Radiation (SRIM) was used to predict the irradiation damage. The SRIM-predicted incident depth profile shows Gaussian distribution (Fig. 2E). The average depth (~404.8 nm) of implanted protons infers that the 40-keV protons can penetrate the prepared AZO nanofibers completely, making it possible to simulate space radiation conditions for testing their robustness in extreme environments. The results of SRIM simulation such as recoil distribution, energy transfer, and distribution of phonons demonstrated that the implanted protons interacted with AZO nanofibers sufficiently (fig. S5).

X-ray diffraction (XRD) patterns of the pristine and irradiated AZO nanofibers show three peaks at 2θ = 31.80°, 34.50°, and 36.32° (Fig. 2F), which correspond to the (100), (002), and (101) crystal planes, respectively. The XRD pattern of the irradiated AZO nanofibers is almost unchanged compared with the pristine sample, revealing that the AZO nanofibers have no structural deterioration after proton irradiation. The optical stability of AZO nanofibers was explored using absorbance spectra and time-resolved photoluminescence (TRPL). First-principles calculations demonstrated that the AZO has a direct bandgap (fig. S6). The absorbance spectra of the pristine and irradiated AZO nanofibers show an absorbance peak (inset of Fig. 2G), with an optical bandgap (*E*_g_) of ~3.27 eV (Fig. 2G) (*23*, *24*). The TRPL curves of AZO nanofibers without and with irradiation show the biexponential decay with the same fitted time constants of 0.4 ns (τ_1_) and 3.29 ns (τ_2_) (Fig. 2H). The mechanisms of the proton irradiation tolerance for AZO nanofibers are as follows: Oxide semiconductors containing Zn, such as zinc oxide (ZnO), have weak bond dissociation energy with oxygen and are generally vulnerable to degradation caused by external stresses (*25*). The Al dopants that act as oxygen binders can reduce the formation of oxygen vacancies, enhance the structural robustness of the metal-oxide lattice, and prevent atomic displacement within the lattice, owing to the relatively high affinity of Al with oxygen atoms (*26*, *27*). On the other hand, semiconductors with a wider bandgap tend to be more radiation robust due to less carrier scattering and charge compensation (*28*). The introduction of Al dopants can widen the bandgap, thus improving the proton radiation resistance (*28*, *29*).

In addition, the XRD pattern of the AZO nanofibers after the thermal cycle treatment remains almost unchanged compared with that before the treatment, demonstrating that the structural characteristics of AZO nanofibers are scarcely affected by the thermal cycle (fig. S7). These results demonstrate that the on-demand printed AZO nanofibers not only exhibit high tolerance to proton irradiation and surpass other irradiation-resistant semiconductors in terms of programmable alignment, irradiation-tolerant duration, and dose (table S1), but also have good thermal cycle stability, which may have potential applications in extreme environments like outer space to enhance the stability and life span of space electronic systems.

### Mimicry of neural polymorphism in cephalopods

**Fig. 3.**
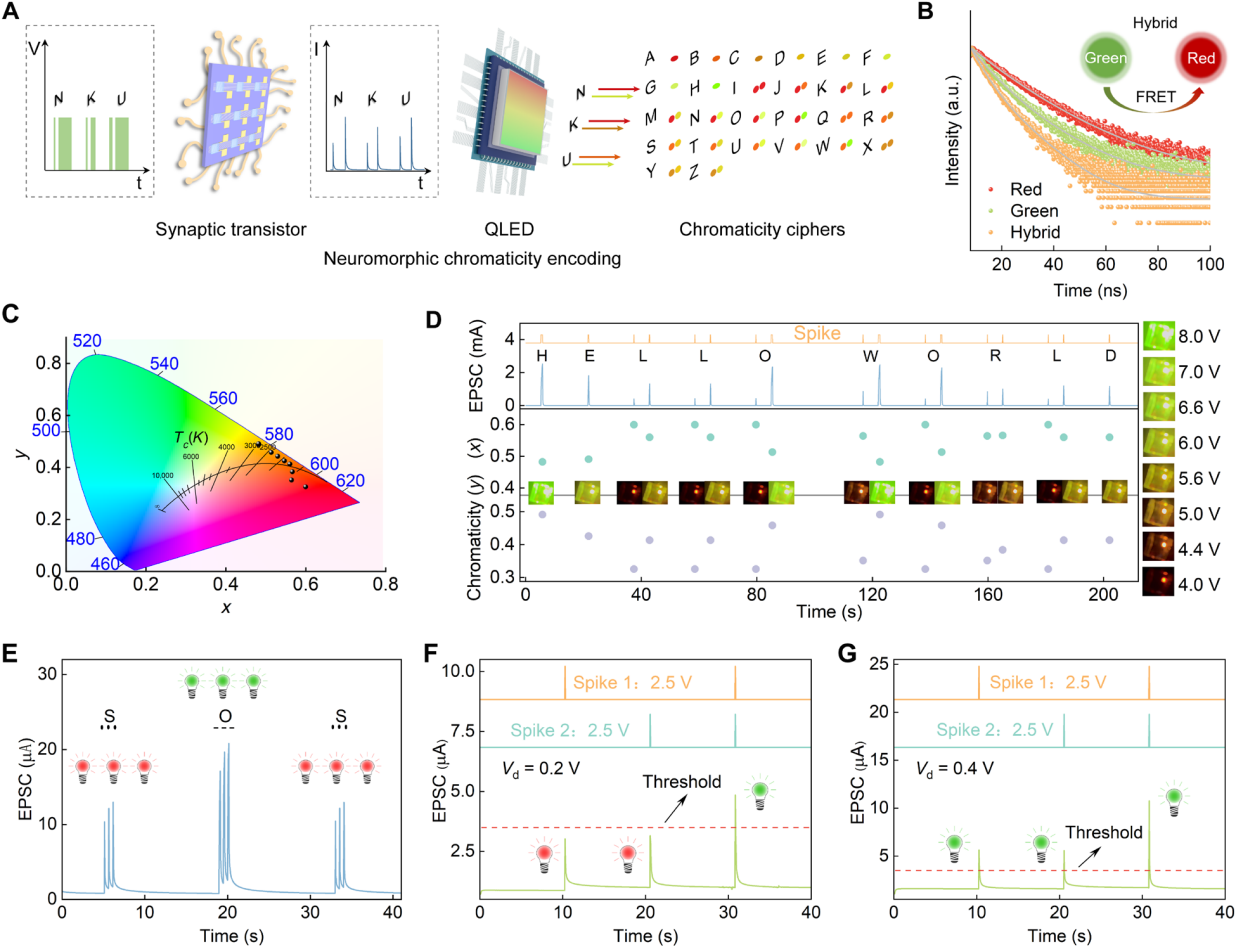
Mimicry of neural polymorphism in cephalopods. (**A**) Schematic of neuromorphic chromaticity encoding inspired by the Humboldt squid. (**B**) TRPL decay of different QDs. Inset: Schematic of the Förster resonant energy transfer between red and green QDs. (**C**) CIE color coordinates of the hybrid QLED under different applied voltages. (**D**) “Hello World” in chromaticity-encoded secure communication. (**E**) Morse code “SOS,” (**F**) logic “AND,” and (**G**) logic “OR” with the corresponding color of light pulses.

Deep-living cephalopods like Humboldt squid use chromatic behaviors that combine complex pigmentation patterning with whole-body bioluminescence for visual communication (*12*). These chromatic communications rely on chromatophore expansion and contraction, driven by motor neuron activity that projects from the brain and forms excitatory glutamatergic synaptic connections with muscles (*30*–*32*). To mimic these neural polymorphisms, we constructed artificial efferent nerves using AFSTs and color-adjustable hybrid quantum dot (QD) light-emitting diodes (QLEDs) to emulate biological synapses and effectors (photonic actuators), respectively (fig. S8). Figure 3A shows a schematic of the neuromorphic chromaticity encoding process. The AFST responds to presynaptic spikes by generating excitatory postsynaptic currents (EPSCs) that are detected by the neuromorphic conversion unit within the peripheral circuits. Subsequently, the circuit emits voltage pulses of varying amplitudes to drive the hybrid QLED, producing light pulses of various colors. The 26 letters of the English alphabet can be encoded in a chromaticity cipher, which endows the artificial efferent nerve with the ability to communicate using language (Fig. 3A). In addition, it can perform neuromorphic displays and logic operations.

AFSTs use an ion gel composed of poly(vinylidene fluoride-co-hexafluoropropylene) (PVDF-HFP) and 1-ethyl-3-methylimidazolium bis-(trifluoromethylsulfonyl)imide (EMIM-TFSI) as the gate dielectric layer. To improve the performance of AFSTs, we incorporated poly(vinyl alcohol) (PVA) mixed with bis-(trifluoromethane) sulfonimide lithium salt (LiTFSI) as a modified layer (fig. S9). The time constants of decay for the AFSTs without the modified layer (AFST-wo) and the AFSTs with the modified layer (AFST-w) were approximately 198 and 188 ms, respectively (fig. S10). The AFSTs have ultrahigh sensitivity (1 mV); however, the AFST-w has higher EPSC, lower threshold voltage (*V*_th_), and larger energy consumption than the AFST-wo (figs. S10 to S12 and table S2). These characteristics can be attributed to the increased amount of cations due to the additional Li^+^; meanwhile, the size of Li^+^ is smaller than the bulky EMIM^+^ in the ion gel (*33*). The smaller Li^+^ migrates more easily under an external electric field and also facilitates a higher packing density at the channel surface, leading to a more efficient electrostatic effect (*34*–*36*). The AFSTs can fully mimic the functions of biological synapses. As spike intervals (Δ*t*) increase from 50 to 4000 ms, the paired-pulse facilitation (PPF) index gradually decreases because of the increased time for cations to diffuse back to their initial distributions after removing the presynaptic spikes (fig. S13). The high-passing characteristics of biological synapses (*37*, *38*), i.e., spike-frequency–dependent plasticity (SFDP), were successfully mimicked and used for image processing (fig. S14). Furthermore, as the amplitude, number, and duration of the presynaptic spike increase, EPSCs are increased monotonically; thus, the spike-voltage–dependent plasticity (fig. S15), spike-number–dependent plasticity (fig. S16), and spike-duration–dependent plasticity (fig. S17) are demonstrated. Our AFSTs exhibit good cycle-to-cycle stability in potentiation/depression under repeated stimuli of positive/negative (2.5 V/−0.3 V) presynaptic spikes (fig. S18). These behaviors indicate that the AFSTs can replicate the synaptic plasticity and stability, making them suitable for information processing and transmission in neuromorphic systems. In addition, the AFSTs were fabricated on glass substrates to investigate their performance before and after proton irradiation of the AZO nanofibers. After proton irradiation, the device did not exhibit obvious deterioration in EPSCs and maintained the ability to emulate biological synaptic plasticity, such as PPF and SFDP (fig. S19).

Using an active region comprising a mixture of different color QDs, QLEDs enable tunable luminous colors within a single device while also offering narrow emission spectra, photophysical stability, and low fabrication costs (*39*, *40*). We fabricated QLEDs using a mixture of green and red CdSe/ZnS QDs with good PL properties (fig. S20 and table S3) to serve as photonic actuators. When two or more separate fluorescent entities are present in suitable proximity (typically <10 nm) and the emission spectrum of one entity (the donor) overlaps the absorption spectrum of another entity (the acceptor), Förster resonance energy transfer (FRET) occurs (*41*, *42*). The TRPL decay times for the red, green, and hybrid QDs are ~13.68, ~9.90, and ~7.00 ns, respectively, and the FRET efficiency of the hybrid QDs is calculated to be ~29.3% (Fig. 3B). Furthermore, the relative flat surface morphologies of the hybrid QDs and ZnO nanoparticle (NP) layers in the hybrid QLED were demonstrated by SEM and AFM images (fig. S21). Electroluminescence (EL) properties of the hybrid QLED, including current density–voltage (*C*-*V*) characteristics, external quantum efficiency, EL spectra, and CIE color coordinates (Fig. 3C and fig. S22), confirm that it meets the requirements as photonic actuators in the artificial efferent nerve. In particular, as the driving voltage increases, the CIE color coordinates shift from red to green region, indicating the tunable luminous colors of a single hybrid QLED. Controlling emission color through single-device multiplexing simplifies circuit complexity and enhances integration.

Twenty-six letters of the English alphabet were successfully represented with chromaticity ciphers through the artificial efferent nerve (figs. S23 to S25). Various words and sentences, such as “Hello World” and “Time Flies,” were successfully encoded (Fig. 3D and fig. S26), suggesting potential applications of this neuromorphic chromaticity encoding strategy in robotics and secure communications. International Morse code, consisting of “dot (.)” and “dash (-),” is successfully generated and displayed in real time using the artificial efferent nerve (Fig. 3E and fig. S27), in which red light pulses as dot (.) and green light pulses as dash (-) (movie S1). For logic operations, green and red light pulses indicate whether the EPSC surpasses or fails to surpass the threshold (3.5 μA), respectively (movie S1). In the execution of logic “AND,” the artificial efferent nerve requires two coincident presynaptic spikes to surpass the threshold, resulting in the emission of green light pulses by the photonic actuator (Fig. 3F). Conversely, a single presynaptic spike is sufficient to surpass the threshold and emit green light pulses when executing logic “OR” (Fig. 3G). Logic operations demonstrate the information processing and intelligence capabilities of the artificial efferent nerve.

### Artificial neural circuit

**Fig. 4.**
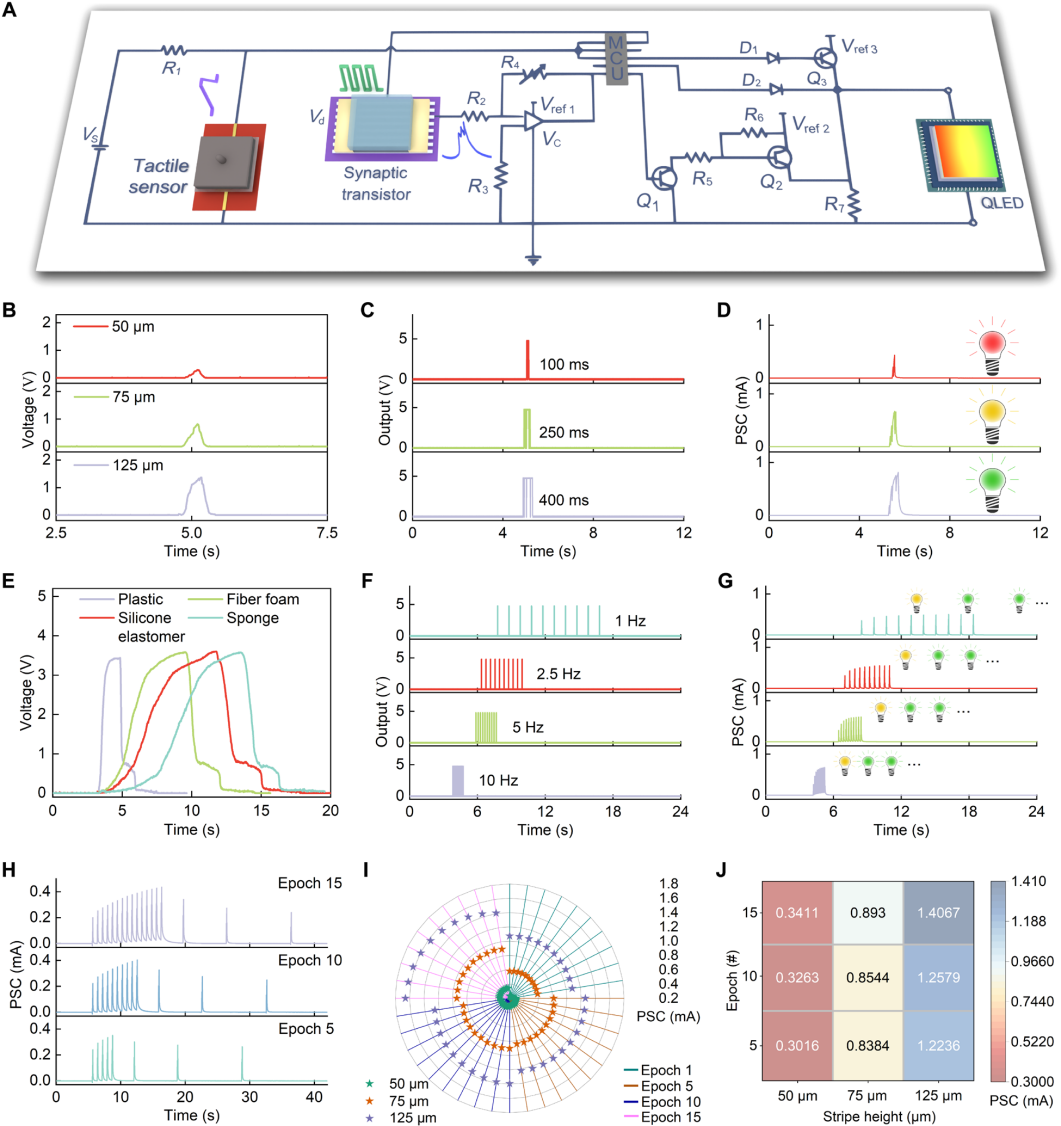
Artificial neural circuit. (**A**) Configuration of the artificial neural circuit. Response of (**B**) the multifunctional tactile sensor, (**C**) output of the neuromorphic coding unit, and (**D**) PSC of the AFST and corresponding color of light pulses when detecting stripes with different heights. Response of (**E**) the multifunctional tactile sensor, (**F**) output of the neuromorphic coding unit, and (**G**) PSC of the AFST and corresponding color of light pulses when detecting materials with different hardness. PSC of the AFST when detecting (**H**) 50-μm stripes and (**I**) stripes with different heights after different training epochs. (**J**) PSC peak of the AFST when detecting stripes with different heights after different training epochs. MCU, microcontroller unit.

Cephalopods use brain lobes to process sensory inputs from the visual, olfactory, and other neurons and then activate motor neurons to control chromatophore expansion and retraction for appearance change (*30*). The neural circuit regulates organ functions to preserve physiological homeostasis, ensuring internal environment stability through reflex arcs that connect sensory inputs to motor outputs (*43*–*45*). We designed a cephalopod-inspired artificial neural circuit including a multifunctional tactile sensor (receptor) and an artificial efferent nerve to link tactile stimulation and optical responses (Fig. 4A and fig. S28). Notably, the tactile sensor can easily switch between horizontal sliding and vertical pressing modes to detect mechanical signals across two dimensions (fig. S29). Mechanical information captured by the tactile sensor is converted by the neuromorphic coding unit in peripheral circuits into presynaptic spikes with distinct duration, number, and frequency, which then evoke EPSCs in the AFST. Subsequently, the neuromorphic conversion unit in the peripheral circuit interprets the EPSCs and generates voltage pulses that match these characteristics, causing the QLED to generate light pulses with various colors, quantities, and frequencies (movie S2).

The artificial neural circuit successfully detects mechanical information in the horizontal dimension, with the tactile sensor in the horizontal sliding mode (Fig. 4, B to D). First, when the artificial neural circuit detects stripes of different heights (50, 75, and 125 μm) (Fig. 4B), presynaptic spikes (5 V) with durations of 100, 250, and 400 ms are applied to the AFST (Fig. 4C), which, in turn, elicit EPSCs of different amplitudes. These EPSCs induce voltage pulses of distinct amplitudes to activate the photonic actuator, emitting light pulses in red, orange, and green, respectively (Fig. 4D). Consequently, the heights of the stripes are successfully distinguished and expressed in different colors. As stripes of varying angles (63.4°, 33.7°, and 26.6°) are sensed (fig. S30A), presynaptic spikes (5 V, 50 ms) in quantities of three, four, and five are applied to the AFST (fig. S30B), which then evoke EPSCs. These EPSCs trigger voltage pulses in quantities of three, four, and five to activate the photonic actuator, emitting light pulses of corresponding numbers that initiate in red and transition to green (fig. S30C). Thus, the angles of the stripes are distinguished and expressed using light pulses of varying quantities. According to the features of stripes, the durations and numbers of the presynaptic spike output from the neuromorphic coding unit can vary in a wide range (fig. S31).

In the vertical pressing mode, the multifunctional tactile sensor enables the artificial neural circuit to detect mechanical information along the vertical dimension (Fig. 4, E to G). For example, the hardness of materials such as sponges, silicone elastomers, fiber foams, and plastics can be distinguished (fig. S32). When these materials are detected (Fig. 4E), 10 presynaptic spikes (5 V, 50 ms) with frequencies of 1, 2.5, 5, and 10 Hz are applied to the AFST to evoke EPSCs (Fig. 4F). Subsequently, these EPSCs trigger voltage pulses with varying frequencies (1, 2.5, 5, and 10 Hz) to activate the photonic actuator and emit light pulses of corresponding frequencies, starting in orange and transitioning to green (Fig. 4G). Notably, when detecting the stripe angle and material hardness, the initial light pulse emitted from the photonic actuator exhibits red and orange colors, respectively, to distinguish different sensing tasks. The maximum power consumption of the artificial neural circuit varies with different tasks being executed, which is approximately 1.109 W for stripe detection and 1.114 W for material hardness detection.

The artificial neural circuit is capable of adaptive learning ability. When detecting stripes with different heights, the neuromorphic coding unit adaptively encodes tactile information into presynaptic spikes with different durations, and the PSC adaptively increased with the increase in the training epoch (Fig. 4H and fig. S33). After training, the PSCs triggered by stimuli are larger than the pretraining, signifying the memory of learned information. As the stimuli interval increases, the PSC gradually decreases, approaching the pretraining level, which exhibits a trend toward forgetting. Notably, as the training epoch increased, the discriminability of these stripes became more pronounced (Fig. 4, I and J, and fig. S34). Furthermore, the PSC gain was defined as *A*_*n*+*x*_/*A*_1_, where *n* is the training epoch, *x* is the number of stimuli after training, and *A*_1_ is the PSC peak before training. PSC gain increases with increasing training epochs and decreases with increasing stimulus intervals (fig. S35). The adaptive learning ability allows neuromorphic systems to continuously learn new information and dynamically adjust their internal parameters during operation. This not only enhances fault tolerance and robustness but also facilitates more effective environmental interaction, thereby promoting the evolution of neuromorphic systems toward greater intelligence and efficiency. Briefly, the artificial neural circuit with adaptive learning ability can perform environmental perception and then convert tactile sensations into optical expression through neuromorphic chromaticity encoding in an event-driven manner.

### Neuromorphic chromaticity communication loop

**Fig. 5.**
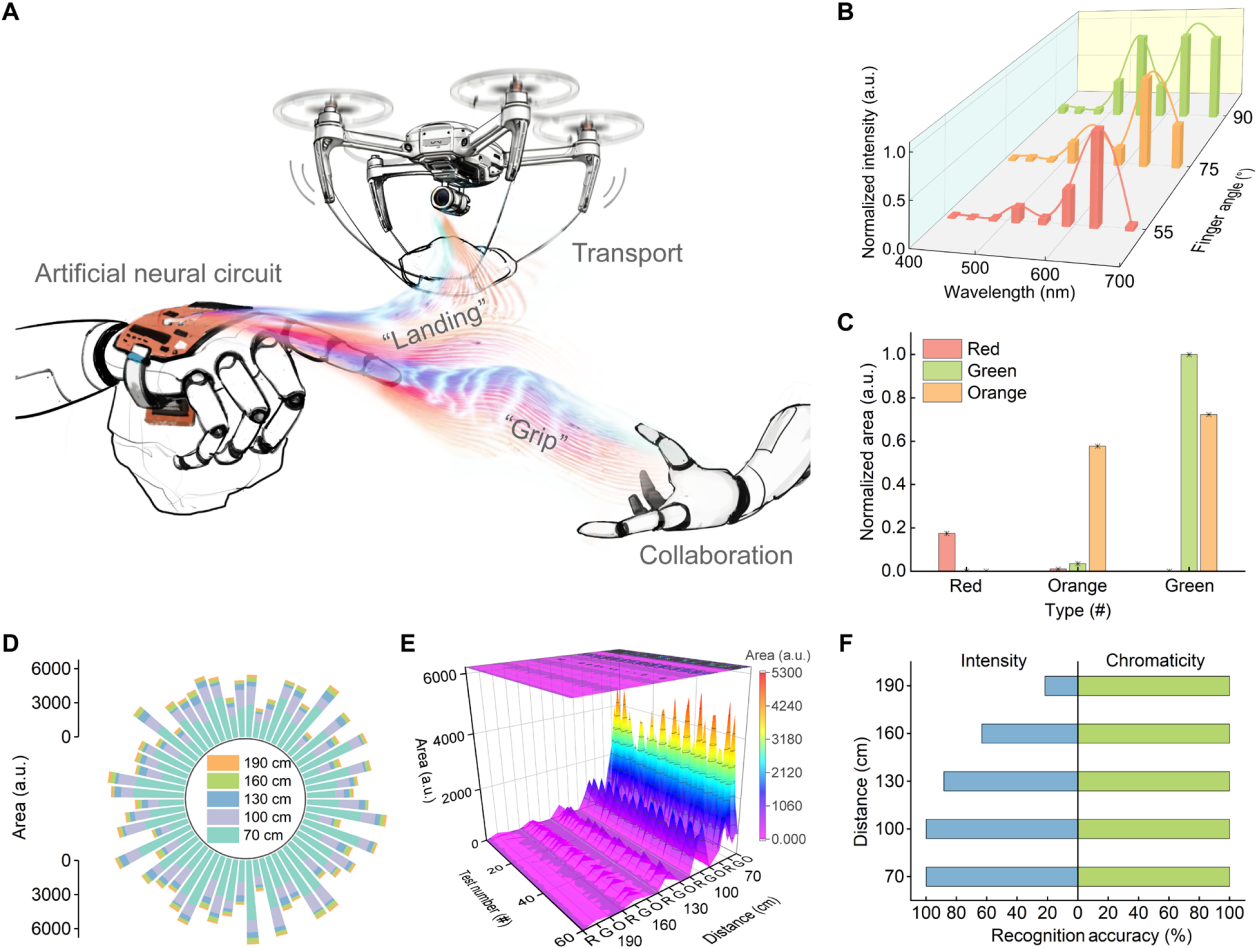
Neuromorphic chromaticity communication loop. (**A**) Schematic of the NCCL in collaboration and transport. (**B**) Light signals emitting from the hybrid QLED collected by the color sensor when detecting stripes with different heights. (**C**) Normalized area of light pulses with varying chromaticity detected by the drone. Emission areas of (**D**) the green luminescent component and (**E**) all luminescent components versus the distance between the QLED and the drone under green light pulses. R, red; G, green; O, orange. (**F**) Comparison of instruction recognition accuracy achieved using intensity and neuromorphic chromaticity encoding strategies.

To implement rapid and efficient group communication, we designed an NCCL that links a transmitter (artificial neural circuit) and a receiver through chromaticity-encoded visible light (Fig. 5A and fig. S36) and conducted a proof of concept for the NCCL. The photodetectors of the receiver detect visual signals emitted from the transmitter and then control the robots (a manipulator and a drone) to execute complex tasks. The introduction of chromaticity expands the density and dimensionality of the perceptible visual information.

As the transmitter senses the stripes with different heights (50, 75, and 125 μm), chromaticity-encoded light pulses emitted from the photonic actuator transform between red, orange, and green in turn, which propagate in free space and are detected by photodetectors in the receiver (Fig. 5B). In response to the color instruction, the fingers of the manipulator folded in different angles to grip the objects with adaptability (fig. S37 and table S4). Similarly, when the transmitter senses the stripes with different angles or the materials with different hardness, the quantities or frequencies of light pulses vary, respectively, which are then detected by the receiver (fig. S38). Under the quantity or frequency instruction, the fingers or palm of the manipulator grip in different states to adapt to different scenarios (figs. S39 to S41 and tables S5 and S6). These results demonstrate that the NCCL is capable of near-sensor processing, neuromorphic chromaticity encoding, and visible light communication (movie S3).

In extreme environments such as space, networked robots need to perceive environmental information and cooperate autonomously in real time to accurately execute complex collective tasks. The NCCL is integrated into a robotic platform, enabling swift response from the manipulator when a hard object is detected, thus facilitating the self-driven collaboration based on tactile sensations (fig. S42A). When the NCCL is mounted on a human hand, it responds with an appropriate grip upon contact with a hard object (fig. S42B), demonstrating the feasibility of applying neuromorphic chromaticity communication to neurorobots and human-machine interaction. The key components within the NCCL can still operate after being placed in an environment without water and oxygen for 3 months (fig. S43). However, their durability still needs to be further improved through the development of functional materials capable of withstanding extreme environments, the fabrication of highly robust electronic devices, and the encapsulation of the electronic devices and systems.

Moreover, the NCCL can be used for drone flight control. Upon detecting green, orange, and red light pulses from the transmitter, the drone executes a full 360° aerial rotation, left-right swaying, and descending actions, respectively (Fig. 5C, figs. S44 and S45, and movie S4). With the increasing distance between the QLED and the drone, the instruction recognition accuracy achieved using the neuromorphic chromaticity encoding strategy was higher than that using the intensity encoding strategy (Fig. 5, D to F). This result demonstrates the advantage of neuromorphic chromaticity encoding strategy in mobile links, implying its potential for smart transportation.

## DISCUSSION

We have demonstrated a cephalopod-inspired NCCL that is primarily composed of a transmitter and a receiver linked via visible light, realizing near-sensor processing, neuromorphic chromaticity encoding, and visible light communication. The artificial neural circuit serving as the transmitter provides the system with environmental perception, event-driven processing, and adaptive learning, allowing tactile sensation–to–optical expression conversion and chromaticity encoding by mimicking cephalopod neural polymorphism. The NCCL facilitates self-driven collaboration of robotic hands based on tactile feedback and offers reliable drone flight control in mobile links. In addition, highly aligned AZO nanofibers were programmatically printed on demand as conductive channels, demonstrating resistance to prolonged (~10^4^ s) and high-dose (~5 × 10^15^ ions/cm^2^) proton irradiation. Our system offers a paradigm for integrating neuromorphic intelligence with optical wireless communication, with potential applications in neurorobots and human-machine interfaces for extreme environments such as space.

## MATERIALS AND METHODS

### Materials

Aluminum nitrate nonahydrate [Al(NO_3_)_3_∙9H_2_O, 99.997%], zinc nitrate hexahydrate [Zn(NO_3_)_2_∙6H_2_O, 98%], and poly(vinyl pyrrolidone) [weight-average molecular weight (*M*_w_), 1,300,000] were purchased from Sigma-Aldrich, Acros, and Innochem, respectively. PVA (*M*_w_, 67,000) and LiTFSI (*M*_w_, 287.09) were purchased from Aladdin. Indium tin oxide (ITO) glass substrates, poly(3,4-ethylenedioxythiophene)–poly(styrenesulfonate) (PEDOT:PSS, PH1000), and poly[bis(4-phenyl)(4-butylphenyl)amine] (Poly-TPD; *M*_w_, 10,000 to 100,000) were purchased from Luoyang Guluo Glass Co. Ltd., Heraeus, and Macklin, respectively. The red and green CdSe/ZnS QDs and ZnO NPs (30 mg/ml in ethanol) were purchased from Poly OptoElectronics Co. Ltd. Multiwalled carbon nanotubes (MWCNTs) with an average length of 10 μm (14 wt % in water), natural latex (solid content, 60%), and adhesive dressing (Tegaderm) were purchased from XF Nano, Shenzhen Shunjie Materials, and 3M, respectively. All these reagents were used without further purification.

### Methods

The Supplementary Materials contains additional experimental details.

### Fabrication of AFSTs

First, a Si/SiO_2_ substrate was cleaned with deionized water, isopropanol, acetone, and anhydrous ethanol, consecutively, for 30 min each. Second, highly aligned AZO nanofibers were printed on the substrate as the channel. Third, PVA and LiTFSI were dissolved in deionized water, forming a mixed solution, and spin coated (2500 rpm, 40 s) onto the AZO nanofibers as a modified layer. Then, gold was thermally deposited through an interdigitated shadow mask as source and drain electrodes (~70 nm). Last, the prepared ion gel was transferred onto the sample as the gate dielectric layer. A metal probe served as the input terminal to apply presynaptic spikes.

### Fabrication of the hybrid QLED

First, ITO glass substrates were sequentially ultrasonicated in deionized water, isopropanol, acetone, and anhydrous ethanol for 20 min each and then treated with ozone for 15 min in air. Second, PEDOT:PSS was spin coated onto the substrates at 3500 rpm for 40 s and baked at 130°C for 15 min in air. Next, except for the top Ag electrode, other functional layers were spin coated in nitrogen-filled glove box. Poly-TPD (in chlorobenzene, 8 mg/ml) and hybrid QDs (in octane, 15 mg/ml) were spin coated layer by layer at 2000 rpm for 45 s and baked at 120°C for 20 min each. Then, ZnO NPs were spin coated at 3000 rpm for 45 s and baked at 120°C for 30 min. Last, the multilayer samples were transferred to a high-vacuum deposition chamber to deposit the Ag electrodes (~120 nm) to form the hybrid QLED with 4 mm^2^ active region area.

### Fabrication of the multifunctional tactile sensor

The mixture of MWCNTs and natural latex (1:1, v/v) was drop cast onto a silicon mold etched with inverse pyramidal structures (size, ~60 μm), which was dried at 50°C for 1 hour. The MWCNTs/latex composite film was peeled from the mold and cut into 3 mm by 3 mm to form elastic sensing layer. Then, the elastic sensing layer was transferred onto a polyimide substrate that has been patterned with interdigitated electrodes (Ti/Au, 5 nm/100 nm). An adhesive dressing was laminated on the top of the elastic sensing layer for encapsulation. The rigid sensing tip (diameter, ~3 mm) was fabricated by stereolithography printing technique using a desktop low-cost 3D printer (Form 3, Formlabs). An additional curing process (ultraviolet light, 30 min, 50°C) was applied to improve the strength and stability of the printed structure made of resin (standard resin made of liquid photopolymer; Formlabs). Last, the rigid sensing tip was assembled onto the surface of the sample using a layer of soft polyurethane foam tape to form the multifunctional tactile sensor. Specifically, the rigid sensing tip serves as the rigid component to effectively detect the mechanical stimuli, the soft foam layer underneath allows the passive deflection of the rigid sensing tip during tactile contact, and the elastic sensing layer with an inverse pyramidal structure converts tactile stimuli into resistance change.

## Supplementary Materials

### The PDF file includes:

Supplementary Text Figs. S1 to S45 Tables S1 to S6 Legends for movies S1 to S4 References
