# Supplementary material for: Oxide semiconductor in a neuromorphic chromaticity communication loop for extreme environment exploration
Source: Sci Adv. 2025 May 16;11(20):eadu3576. doi: 10.1126/sciadv.adu3576 (PMC12083538; doi:10.1126/sciadv.adu3576)
Supplement: Supplementary file 1 — Supplementary Text Figs. S1 to S45 Tables S1 to S6 Legends for movies S1 to S4 References [file sciadv.adu3576_sm.pdf]

Science Advances
AAAS

# Supplementary Materials for

## Oxide semiconductor in a neuromorphic chromaticity communication loop for extreme environment exploration

Shangda Qu *et al.*

Corresponding author: Wentao Xu, wentao@nankai.edu.cn; Yong-Young Noh, yynoh@postech.ac.kr

*Sci. Adv.* **11**, eadu3576 (2025)
DOI: 10.1126/sciadv.adu3576

**The PDF file includes:**

Supplementary Text
Figs. S1 to S45
Tables S1 to S6
Legends for movies S1 to S4
References

**Other Supplementary Material for this manuscript includes the following:**

Movies S1 to S4

## Supplementary Text

### Supplementary Method 1. Preparation of AZO nanofibers

$Al(NO_3)_3{\cdot}9H_2O$, $Zn(NO_3)_2{\cdot}6H_2O$, and PVP were dissolved in *N,N*-Dimethylformamide (DMF, 99.8%) and then stirred at 50 °C for 12 h to form printing ink. In the ink, the molar ratio of Al:Zn was fixed to 5:95. The highly-aligned AZO nanofibers were digitally printed on $Si/SiO_2$, glass, or Si substrates using an electrohydrodynamic nanowire printer. In the printing process, the feed rate of the ink, the voltage applied to the nozzle, the nozzle tip-to-collector distance, and the movement speed of the collector were set as ~30 nL/min, ~1.15 kV, ~3.5 mm, and ~1000 mm/s, respectively. After printing, the samples were annealed in a muffle furnace for 2 h at 500 °C.

### Supplementary Method 2. The process of proton irradiation for AZO nanofibers

The proton irradiation experiment on AZO nanofibers was carried out at the National Space Science Center. The proton irradiation facility is mainly composed of a vacuum tank with 63 cm height and 60 cm diameter, a proton flying tube, and a low-energy proton source. The samples were vertically set inside the vacuum tank. The proton irradiation was performed using the 40 keV proton beams with a flux of $5 \times 10^{11}$ $cm^2/s$ and a dose of $5 \times 10^{15}$ $ions/cm^2$ in vacuum $1 \times 10^{-4}$ Pa at room temperature.

### Supplementary Method 3. Preparation of the ion gel

The AZO nanofibers-exploiting synaptic transistor utilized ion gels as the gate dielectric layer. Firstly, poly(vinylidene fluoride-co-hexafluoropropylene) (PVDF-HFP), 1-ethyl-3-methylimidazolium bis-(trifluoromethylsulfonyl)imide ([EMIM-TFSI]), and acetone (1:4:7, w/w/w) were mixed at room temperature. Then, the mixed solution was dried in a vacuum oven at 70 °C for 30 min to form the ion gels.

### Supplementary Method 4. Construction of the artificial efferent nerve inspired by the Humboldt squid

The artificial efferent nerve consists of an AZO nanofibers-exploiting synaptic transistor, a peripheral circuit, and a hybrid QLED. The EPSCs output by the synaptic transistor are collected by a microcontroller that controls the power unit to output voltage pulses with different amplitudes (3 ~ 10 V) relying on the EPSCs values. The output from the power unit drives the hybrid QLED to emit light pulses of different colors.

### Supplementary Method 5. Construction of the artificial neural circuit

The artificial neural circuit consists of a multifunctional tactile sensor, an AZO nanofibers-exploiting synaptic transistor, a peripheral circuit, and a hybrid QLED. The multifunctional tactile sensor can conduct surface texture detection and material hardness discrimination, operating in horizontal sliding mode and vertical pressing mode, respectively. One chip of a microcontroller acts as the neuromorphic coding unit to transform the sensor signal into presynaptic spikes with different durations, numbers, or frequencies. One output terminal of the microcontroller is connected to the gate of the AZO nanofibers-exploiting synaptic transistor to apply presynaptic spikes that trigger EPSCs. The EPSCs output from the synaptic transistor are detected by another chip of the microcontroller that controls the power unit to output voltage pulses with different amplitudes (3 ~ 10 V), numbers, and frequencies relying on the EPSCs. The output of the power unit drives the hybrid QLED to emit light pulses of different colors, numbers, or frequencies, realizing the conversion from tactile sensation to optical expression. The power consumption of

the tactile sensor, synaptic transistor, and QLED is approximately 2.33 ~ 13.49 mW, 0.22 ~ 0.41 mW, 8.42 ~ 850.26 mW, respectively. The power consumption of the microcontroller is 250 mW.

**Supplementary Method 6. Construction of the neuromorphic chromaticity communication loop (NCCL)**

The NCCL is mainly composed of a transmitter and a receiver, which interact via visible light. The transmitter employed the above artificial neural circuit. The receiver employed robots (a manipulator and a drone) equipped with photodetectors. For the receiver, the photodetectors collect visual signals emitting from the transmitter, and then actuate the robots to respond. The manipulator (STM32F103RBT6, STMicroelectronics) equips with a color sensor (AS7341, Waveshare) and a photosensitive resistor (GL5516, Senba Sensing Technology). A microcontroller built into the manipulator is used to demodulate the collected light signals and actuate the manipulator meanwhile. The drone (F450-4B, Free Flight DIY) equips with Raspberry Pi 4B. The average time taken from sensing the tactile signal to the receiver's response is approximately 1 ~ 2.5 s for the NCCL, which varies with the increase in the complexity of tactile sensor inputs. The response time of the NCCL represents a significant improvement and advantage compared to the delays caused by communication between Earth and other planets. For instance, the dual-way communication delay between the Earth and Mars is approximately 360 s. The power consumption of the manipulator, color sensor, photosensitive resistor, and drone is approximately 10, 0.198, 0.09, and 600 W, respectively. The power consumption of communication using chromaticity-encoded signals per event is approximately 206.42 ~ 1048.26 mW. For the entire NCCL system, the maximum power consumption required to complete the manipulator control task and the drone flight control task is approximately 11.40 and 601.11 W, respectively. The majority of the power consumption for task execution is attributed to the commercial manipulator and drone. Therefore, the power consumption of the entire NCCL system can be improved by selecting more energy-efficient manipulators and drones.

**Supplementary Method 7. Material characterization and device measurements**

The morphology of AZO nanofibers, hybrid QDs, ZnO NPs, and MWCNTs/latex composite film were observed using a scanning electron microscope (SEM) (Apreo S, Thermo Scientific). Atomic force microscopy (AFM) images of AZO nanofibers, hybrid QDs, and ZnO NPs were obtained using a Bruker dimension icon microscope in tapping mode. A high-resolution transmission electron microscope (HRTEM) (JEM-2800, JEOL) was used to collect the HRTEM and elemental mapping images of AZO. X-ray photoelectron spectroscopy (XPS) images of AZO nanofibers were obtained using an ESCALAB 250Xi instrument (Thermo Scientific). X-ray diffraction (XRD) patterns of AZO nanofibers were obtained using a Rigaku Ultima-IV instrument. Absorption spectra of AZO nanofibers were obtained using an ultraviolet–visible-near infrared (UV–Vis-NIR) spectrophotometer (Cary5000, Agilent). Fourier transform infrared spectroscopy (FTIR) was obtained using a Nicolet iS20 instrument (Thermo Scientific). Photoluminescence (PL) and time-resolved photoluminescence (TRPL) spectra of AZO nanofibers and QDs were obtained using a fluorescence spectrometer (FS5, Edinburgh). Electroluminescence (EL) characterization of the hybrid QLED was performed using a Keithley 2400 source meter coupled with a fiber spectrometer (FOIS-1-FL integration sphere and QE65 Pro, Ocean Optics). A semiconductor parameter analyzer (B1500A, Agilent) was used to characterize the frequency-dependent capacitance of the gate dielectric layer. All electrical measurements of AZO nanofibers-exploiting

synaptic transistors were conducted using a semiconductor parameter analyzer (4200A, Keithley) and a probe station in an $N_2$ environment at room temperature.

**Supplementary Note 1. First-principles calculations**

The Vienna Ab-initio Simulation Package (VASP) was employed to conduct first-principles calculations that apply density functional theory (DFT) (*20, 21*). According to the generalized gradient approximation (GGA) using Perdew-Burke-Ernzerhof (PBE) functions, the projector-augmented wave (PAW) pseudopotentials were used to describe the interaction between the valance electrons and ionic cores (*46, 47*). In this calculation, hexagonal wurtzite was employed as the initial structure of AZO. To obtain the molar ratios of Al:Zn (5:95), a 5 × 4 × 1 supercell was built using the special quasi-random structure (SQS) approach (*48, 49*), in which two Zn atoms were substituted with Al atoms. The cut-off energy for the wave function and the convergence criterion for self-consistent calculation were set as 500 eV and $10^{-6}$ eV, respectively. The Brillouin zone was sampled by using a Γ-centered mesh with a sampling density of 0.02 × 2π $Å^{-1}$. All atoms were fully relaxed until all the residual forces were < 0.001 eV/Å. The Vaspkit code was used to conduct data post-processing and band unfolding (*50*).

**Supplementary Note 2. SRIM simulation**

Radiation damage to the AZO nanofibers was modeled using the theoretical Monte Carlo simulation software package Stopping and Range of Ions in Matter (SRIM)-2013, which can calculate various features of ions transport in matter. The main parameters used in the simulation were as follows: Ion = $H^+$, Ion mass = 1.0080, Energy = 40 keV, and Ion angle to surface = 0 degrees. The target consisted of three layers: AZO (~ 200 nm), $SiO_2$ (~ 300 nm), and Si (~ 2.5 μm), respectively.

**Supplementary Note 3. Calculation of optical band gap of AZO nanofibers**

The optical band gap ($E_g$) of AZO nanofibers was calculated according to the following equation (*23, 24*):

$$\alpha h\upsilon = \mathrm{A}\left(h\upsilon - E_g\right)^n$$

where $\alpha$, $h$, $v$, and A are the absorption coefficient, Planck's constant, photon's frequency, and constant, respectively. Here, $n = 0.5$.

**Supplementary Note 4. Image processing**

An image of a building was extracted and processed using Python. The image was transformed to grayscale mode from RGB mode and then converted to frequency domain from spatial domain by Fourier transform. After that, the zero-frequency component was moved to the center to rearrange the matrix in the frequency domain. Then, an $m \times n$ filter template was proposed according to the new matrix. A high-pass filter was established based on the filtering behaviors of the AZO nanofibers-exploiting synaptic transistor with a modified layer. In the filter template, $f$ is the Euclidean distance between the center ($u = 0.5\ m$, $v = 0.5\ n$) and a specific point $(i, j)$, where $f^2 = (i - u)^2 + (j - v)^2$, $f_c$ is the cut-off frequency of the filter. A matrix $G = H\ (i, j)\ N\ (i, j)$ was utilized for filtering, which was decentralized and transformed to a matrix in the spatial domain by inverse Fourier transform to obtain the ultimate result.

**Supplementary Note 5. Calculation of Förster resonant energy transfer efficiency**

Firstly, carrier lifetimes of red, green, and hybrid QDs were acquired by bi-exponential fitting using equation (1) listed below (*51, 52*). Here, red and green QDs are acceptor QDs and donor QDs, respectively.

$$\boldsymbol{I}_t = \alpha_1 e^{\frac{-t}{\tau_1}} + \alpha_2 e^{\frac{-t}{\tau_2}} \qquad (1)$$

Then, the Förster resonant energy transfer efficiency ($\eta_{\mathrm{FERT}}$) carrier was calculated using carrier lifetimes of donor QDs through equation (2) (*53*).

$$\boldsymbol{\eta}_{\mathrm{FERT}} = 1 - \frac{\tau_{\mathrm{DA}}}{\tau_{\mathrm{D}}} \qquad (2)$$

where $\tau_{\mathrm{DA}}$ and $\tau_{\mathrm{D}}$ are the average fluorescence lifetimes of the donor QDs in the presence and absence of acceptor.

**Supplementary Note 6. Neuromorphic chromaticity encoding inspired by the neural polymorphism of the Humboldt squid**

Eight light pulses with distinct chromaticity were utilized for neuromorphic chromaticity encoding, which were emitted by QLEDs controlled by a synaptic transistor. Application of presynaptic spikes (5 V) with varying durations (50, 100, 150, 200, 300, 400, 500, and 600 ms) to the synaptic transistor triggered EPSCs with different amplitudes. Subsequently, corresponding voltage pulses of 4.0, 4.4, 5.0, 5.6, 6.0, 6.6, 7.0, and 8.0 V were applied to the QLEDs to emit light pulses with varying chromaticity. These chromaticity are sequentially defined as 1 to 8, representing English letters A to H, respectively. Two consecutive light pulses with combined chromaticity of 11, 12, 13, 14, 15, 16, 17, 18, 22, 23, 24, 25, 26, 27, 28, 33, 34, and 35 are defined as I~Z, respectively. Thus, the 26 letters of English alphabet can be represented by chromaticity. Furthermore, different words and sentences can be represented relying on neuromorphic chromaticity encoding strategy.

**Supplementary Note 7. Generation of international Morse code**

International Morse code consists of “dot (.)” and “dash (-)”. The EPSCs smaller than and larger than a threshold are defined as “dot (.)” and “dash (-)”, respectively, which are triggered by short-duration presynaptic spikes (2.5 V, 50 ms) and long-duration presynaptic spikes (2.5 V, 150 ms) at drain voltage of 0.4 V. The threshold is defined as 15 μA for the AZO nanofibers-exploiting synaptic transistor with a modified layer and as 7.5 μA for the AZO nanofibers-exploiting synaptic transistor without a modified layer.

**Supplementary Note 8. Dynamic logic operation**

Dynamic logic operations primarily involve logic “AND” and logic “OR”. Here, the threshold is defined as 3.5 μA. Presynaptic spikes (2.5 V, 50 ms) are applied to the AZO nanofibers-exploiting synaptic transistor without a modified layer by two gate electrodes. Only when the EPSC triggered by two concurrent presynaptic spikes can surpass the threshold, it corresponds to logic “AND”. Conversely, when the EPSC triggered by a single presynaptic spike can surpass the threshold, it corresponds to logic “OR”. To achieve “AND” and “OR” logic operation, the drain voltage of the synaptic transistor is set to 0.2 V and 0.4 V, respectively.

**Supplementary Note 9. Heights and angles detection of stripes**

Stripes of different heights (50, 75, and 125 μm) and angles (26.6, 33.7, and 63.4°) were prepared using a 3D printer (Form 3, Formlabs). Here, the angle of a stripe refers to the angle formed between the height of the inclined stripe and its base edge. During detection, the objects were fixed

on the Mark-10 digital force gauges (Series 7) and moved at a speed of 3 mm/s to slide across the surface of the multifunctional tactile sensor. The rigid sensing tip of the multifunctional tactile sensor contacted these measured objects.

**Supplementary Note 10. Hardness detection of different materials**

The materials detected included sponge, silicone elastomer, fiber foam, and plastic. For detection, these materials were mounted on the Mark-10 digital force gauges (Series 7) and moved at a speed of 0.5 mm/s to depress the multifunctional tactile sensor. The rigid sensing tip of the sensor made contact with these measured materials.

**Supplementary Note 11. Drone flight control via chromaticity-encoded visible light communication**

The QLEDs are controlled by the synaptic transistors to emit green, orange, and red light pulses. Upon detecting these light pulses, the drone executes various flight maneuvers to adjust its flying posture. Specifically, when a green light pulse is detected, the drone rotates in mid-air; upon sensing an orange light pulse, the drone undergoes left-right oscillation and then returns to its original position; and when a red light pulse is detected, the drone initiates a descent. Consequently, the drone flight can be manipulated through chromaticity-encoded visible light communication.

## Supplementary Figures

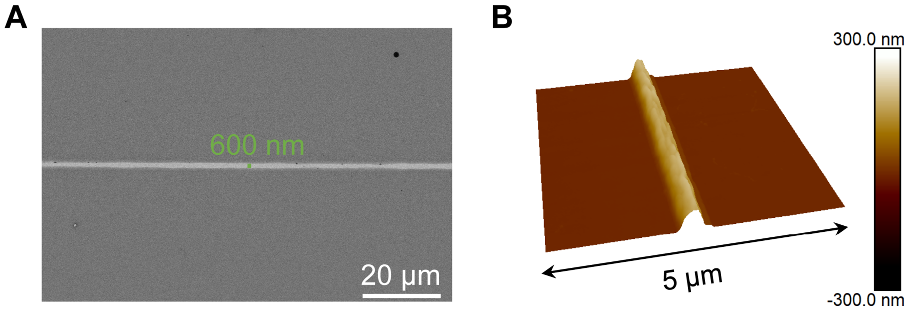


**Fig. S1. Surface morphology of a single AZO nanofiber.** (**A**) SEM image. (**B**) AFM image.

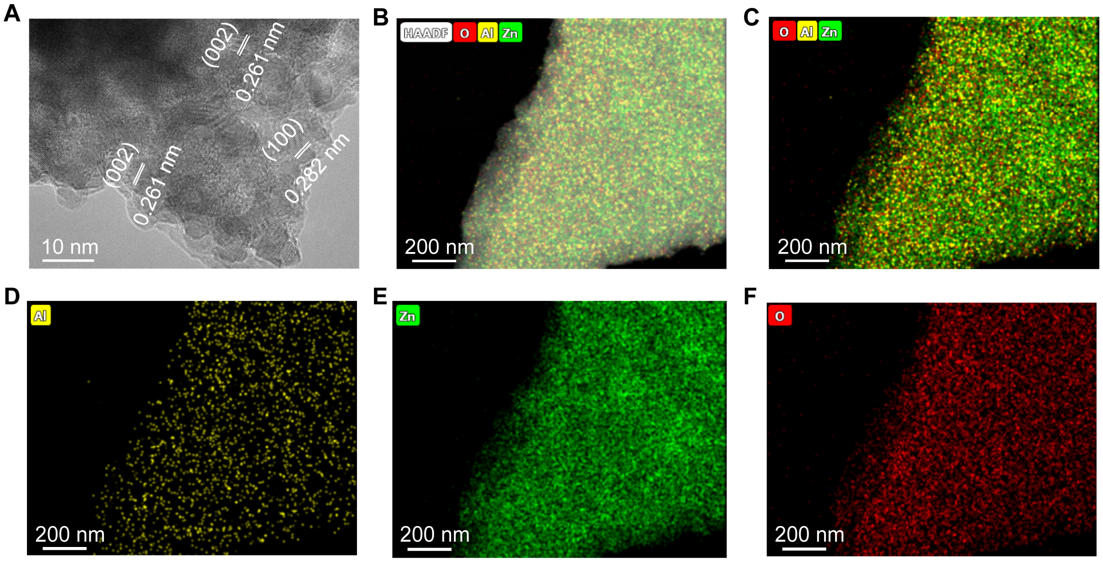


**Fig. S2. HRTEM, high-angle annular dark-field scanning TEM (HAADF-STEM), and elemental mapping images of AZO nanofibers.** (**A**) HRTEM image. (**B**) HAADF-STEM image. Elemental mapping images of (**C**) AZO, (**D**) Al, (**E**) Zn, and (**F**) O.

Note: In the HRTEM image, lattice fringes with spacings of ~0.282 nm and ~0.261 nm are observed clearly, which are assigned to (100) and (002) crystal planes of AZO nanofibers, respectively.

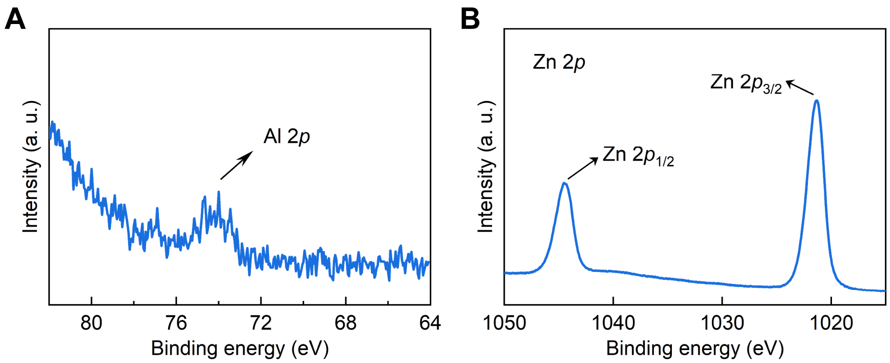


**Fig. S3. XPS spectra of the AZO nanofibers**. (**A**) Al 2*p*. (**B**) Zn 2*p*.

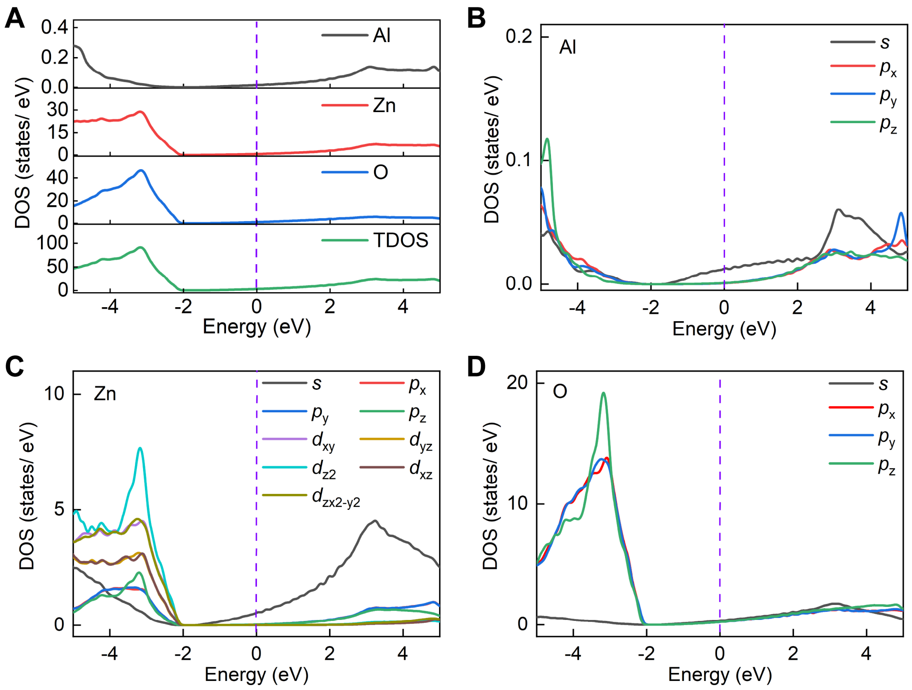


**Fig. S4. Density of states and partial density of states for AZO.** (**A**) Density of states for AZO. Partial density of states for (**B**) Al, (**C**) Zn, and (**D**) O.

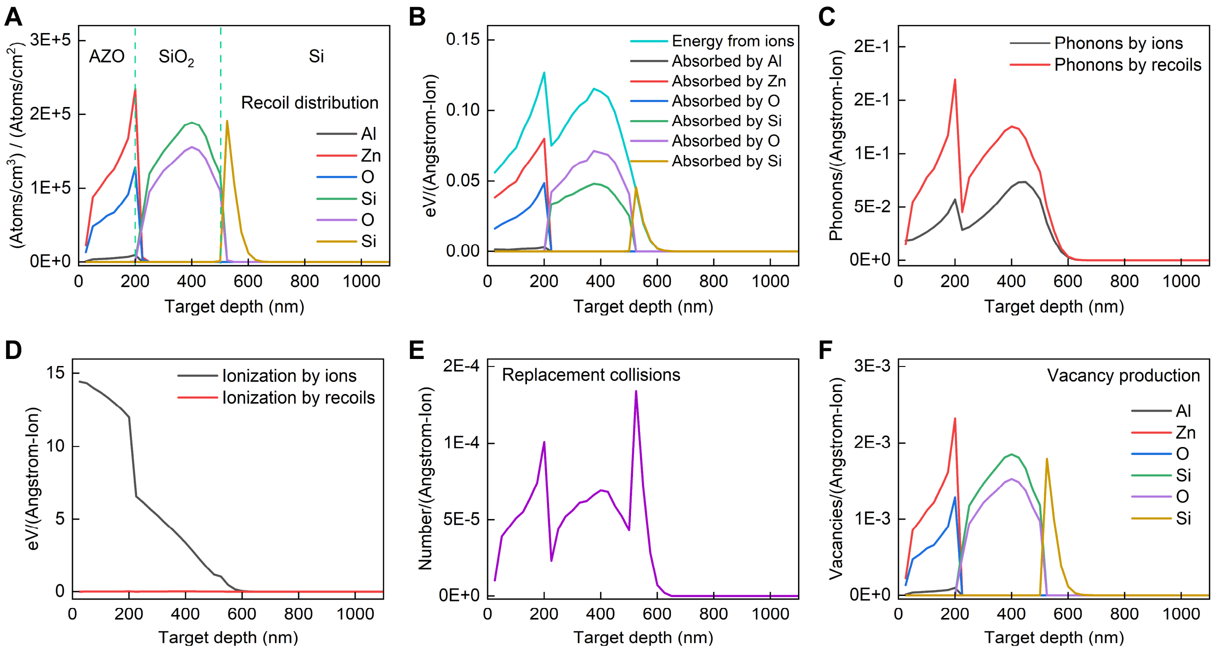


**Fig. S5. Results of SRIM simulation**. (**A**) Recoil distribution. (**B**) Energy transferred to recoils. (**C**) Distribution of phonons. (**D**) Distribution of ionization. (**E**) Replacement collisions. (**F**) Distribution of vacancies.

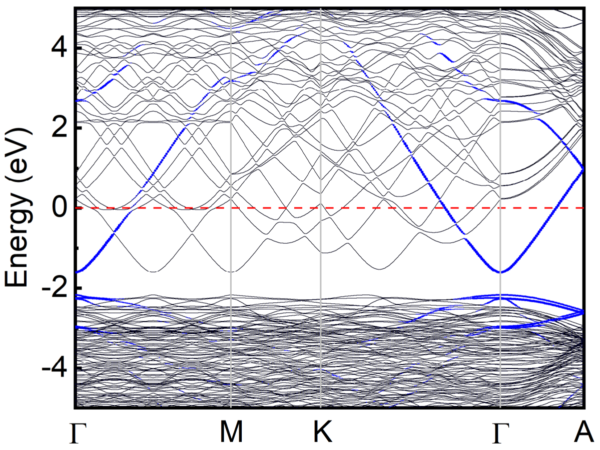


**Fig. S6. Band structure of AZO with a 5 × 4 × 1 supercell.** The size of the blue circles indicates the weight of the unfolded band structure.

Note: The band structure was calculated through the band unfolding method, with its main dispersion reproduced and colored in blue. Owing to the alignment of the valence band maximum and the conduction band minimum at the Γ point, AZO possesses a direct band gap.

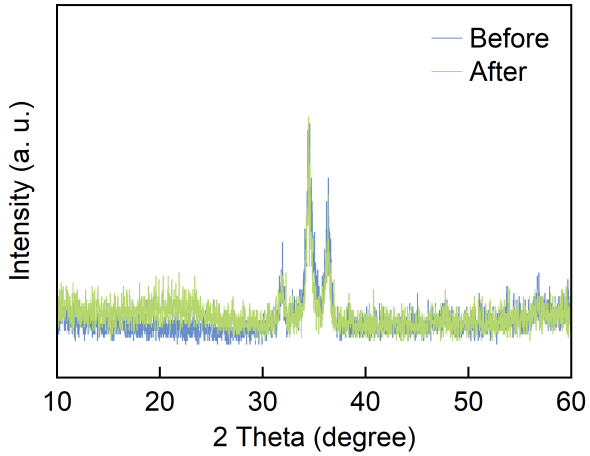


**Fig. S7. XRD patterns of the AZO nanofibers before and after the thermal cycle treatment.**

Note: The sample was subjected to more than 40 thermal cycle treatments within the temperature range from 60 ℃ to -60 ℃ using the programmable temperature chamber.

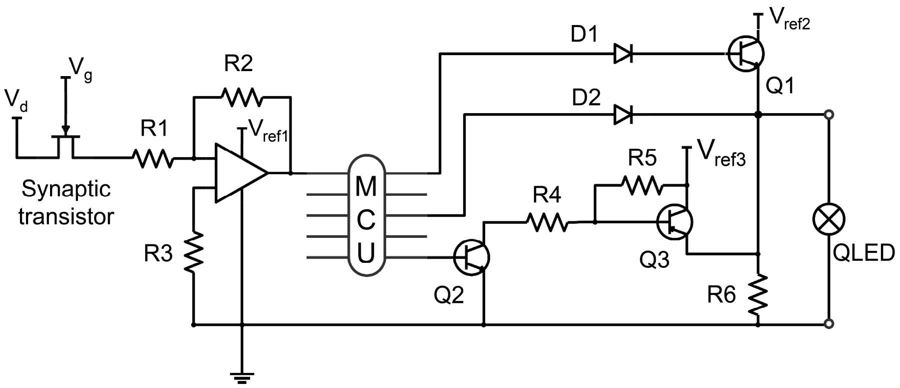


**Fig. S8. Schematic diagram of the integrated circuit for the artificial efferent nerve inspired by the Humboldt squid.**

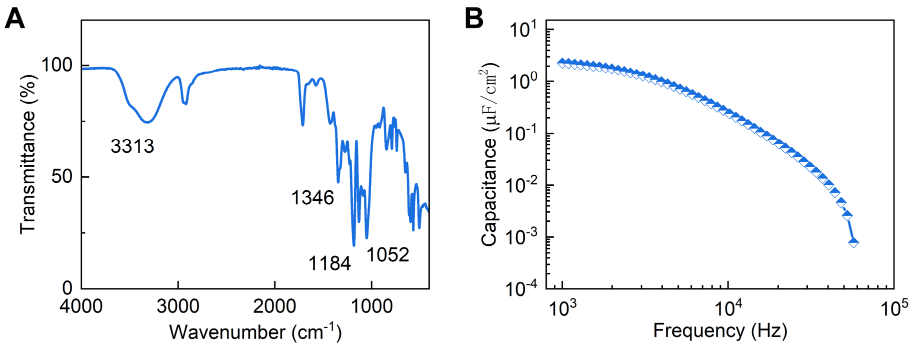


**Fig. S9. Characterization for the PVA:Li and the dielectric layer.** (**A**) FTIR spectrum of the PVA:Li. (**B**) Frequency-dependent capacitance of the dielectric layer.

Note: AFSTs utilize AZO nanofibers as channels, poly(vinyl alcohol) (PVA) mixed with bis-(trifluoromethane) sulfonimide lithium salt (LiTFSI) as a modified layer (with or without), and ion gels as the gate dielectric layer. The peak at ~3313 $cm^{-1}$ is assigned to –OH, and the peaks at 1346, 1184, and 1052 $cm^{-1}$ correspond to $TFSI^{-}$ (fig. S9A). The frequency-dependent capacitance of the dielectric layer is ~2.29 $\mu F/cm^2$ at 1 kHz (fig. S9B).

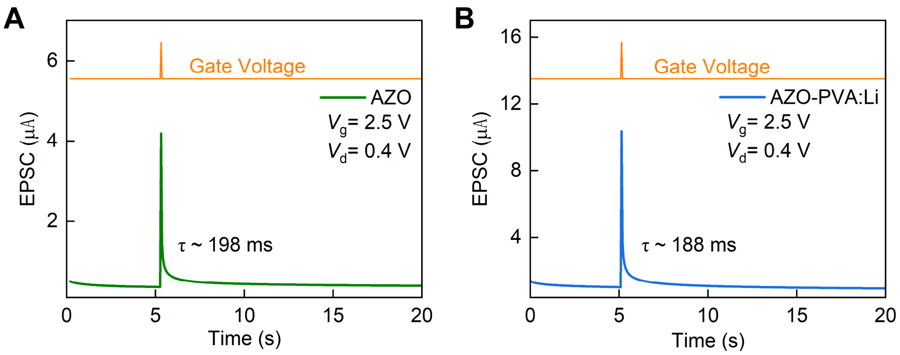


**Fig. S10. EPSC of the AZO nanofibers-exploiting synaptic transistors triggered by a single spike.** (**A**) Without a modified layer. (**B**) With a modified layer.

Note: The EPSCs of the AFSTs with and without a modified layer were measured by applying a single presynaptic spike (2.5 V, 50 ms) to the gate at drain voltage ($V_d$) = 0.4 V. The cations in the ion gel and modified layer drift to the surface of AZO nanofibers due to the positive spike, resulting in the accumulation of electrons in the channel surface and the formation of an electric double layer caused by the electrostatic effect. As a result, the channel conductance increases and yields EPSC. The EPSC decay process was fitted using the following equation (*54*):

$$\boldsymbol{I}_t = \mathrm{A} \cdot e^{\frac{-t}{\tau}} + I_0$$

where A, $\tau$, and $I_0$ are pre-exponential factor, time constant of decay, and quiescent current after EPSC decay, respectively. The $\tau$ values of the AFSTs without and with a modified layer were approximately 198 and 188 ms, respectively.

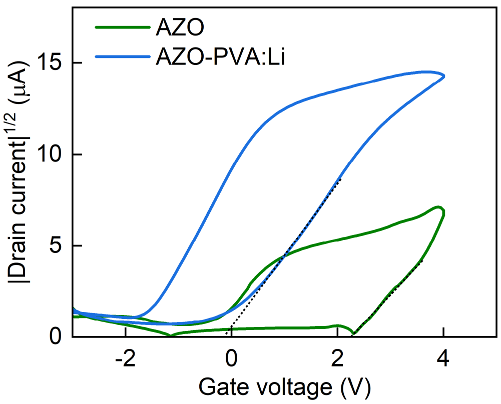


**Fig. S11. |Drain current|$^{1/2}$ as a function of gate voltage (from -3 to 4 V) for AZO nanofibers-exploiting synaptic transistors at drain voltage $V_d$ = 0.4 V.**

Note: The transfer characteristic of the AFSTs demonstrated that the channel conductance can be effectively tuned, with the AFST-w exhibiting a lower threshold voltage ($V_{th}$) than the AFST-wo.

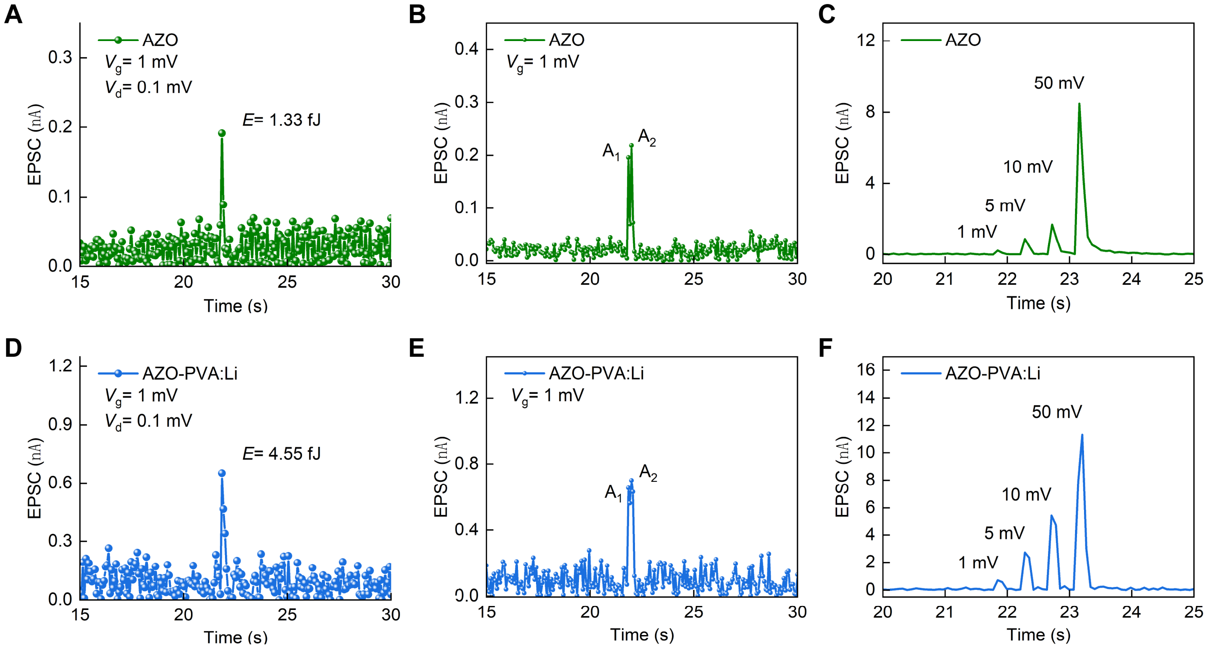


**Fig. S12. EPSC of AZO nanofibers-exploiting synaptic transistors triggered by a single spike (1 mV, 50 ms), a pair of spikes (1 mV, 50 ms), and spikes (50 ms) with amplitudes 1, 5, 10, or 50 mV.** (**A**), (**B**), (**C**) Without a modified layer. (**D**), (**E**), (**F**) With a modified layer.

Note: Energy consumption of AFSTs was calculated via $E = AIW$, where $A$, $I$, and $W$ are drain voltage, current, and duration of the spike, respectively (*55*). The minimum $E$ of the AFST-wo and AFST-w under operation voltage of 1 mV are ~1.33 and ~4.55 fJ per synaptic event, respectively.

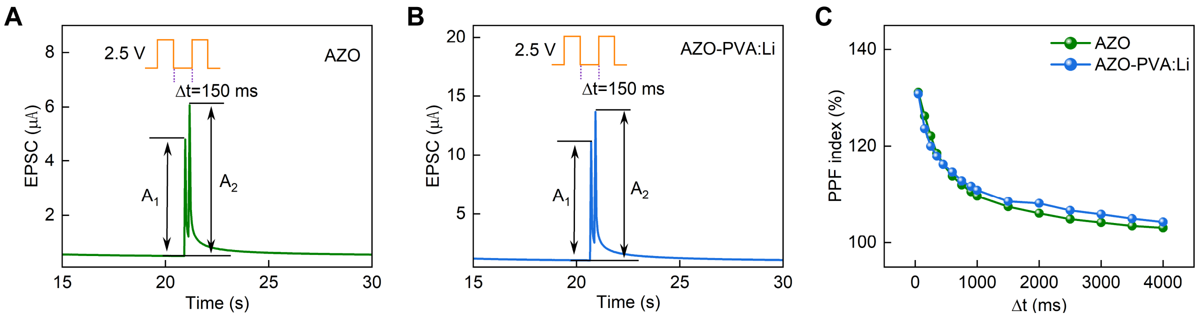


**Fig. S13. EPSC triggered by a pair of spikes (2.5 V, 50 ms) at $V_d$ = 0.4 V for AZO nanofibers-exploiting synaptic transistors.** (**A**) Without a modified layer. (**B**) With a modified layer. (**C**) PPF index.

Note: PPF was successfully mimicked using AFSTs by applying a pair of presynaptic spikes (2.5 V, 50 ms) with different spike intervals (Δ*t*) at $V_d$ = 0.4 V. When Δ*t* = 150 ms, the EPSC peak trigger by the second spike ($A_2$) is higher than that trigger by the first spike ($A_1$) (fig. S13, A and B). Here, PPF index is defined as $A_2/A_1 \times 100\%$ (fig. S13C).

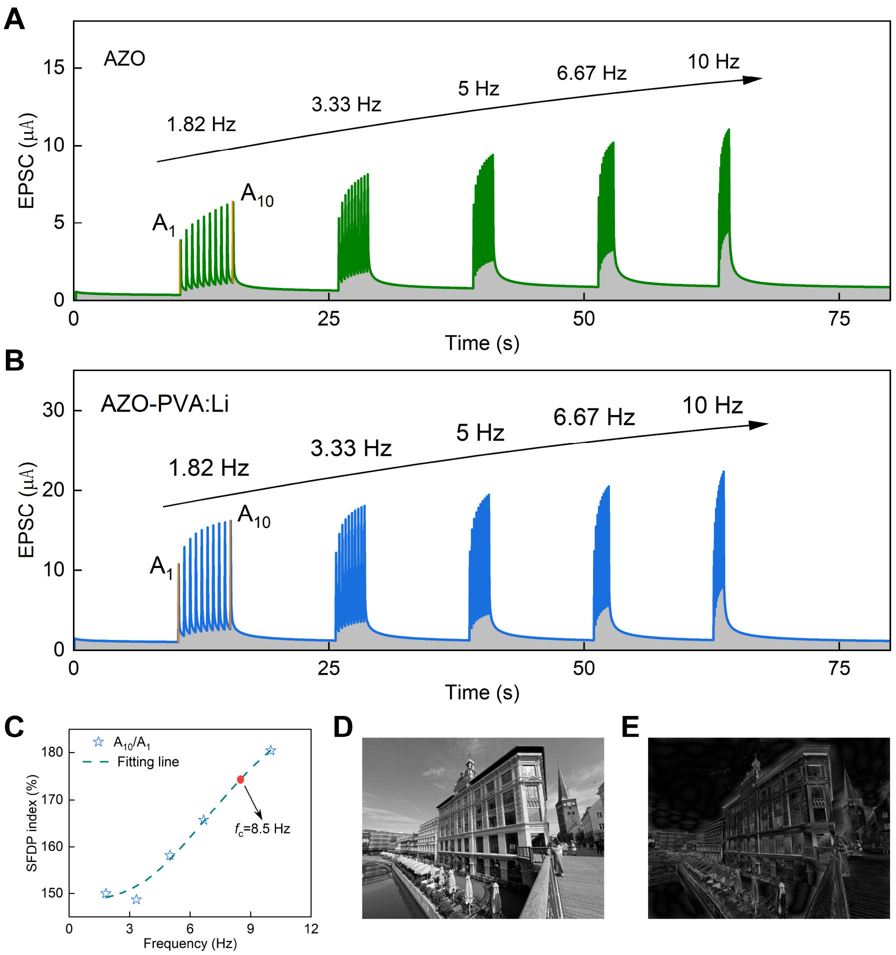


**Fig. S14. SFDP and image processing capacity of the AZO nanofibers-exploiting synaptic transistors.** (**A**) EPSC of the AFST-wo and (**B**) AFST-w triggered by ten consecutive spikes (2.5 V, 50 ms) with spike frequencies 1.82, 3.33, 5, 6.67, and 10 Hz at $V_d = 0.4$ V. (**C**) Cut-off frequency ($f_c$) fitted from the SFDP index for the AFST-w. Images (**D**) before and (**E**) after processing.

Note: Here, the SFDP index is defined as $A_{10}/A_1 \times 100\%$. Based on the SFDP, the AFST-w was used for image processing. The edges of buildings are sharpened after processing, and the $f_c$ fitted from the SFDP index is ~8.5 Hz.

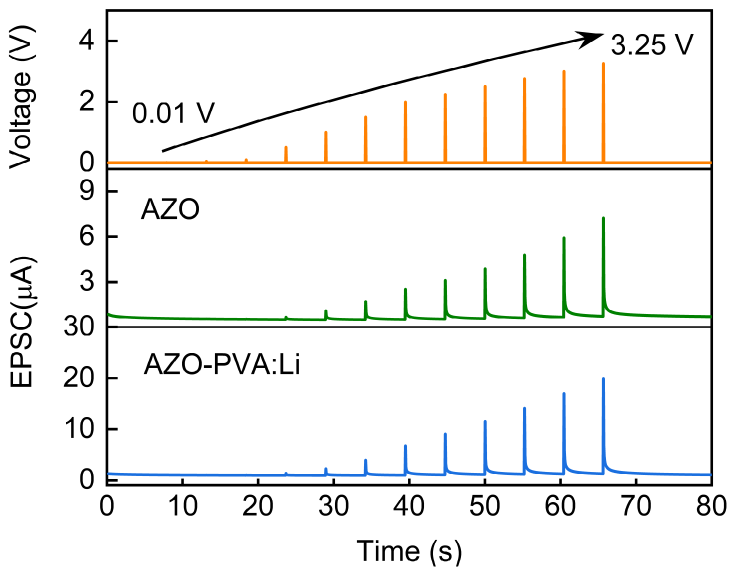


**Fig. S15. EPSC of AZO nanofibers-exploiting synaptic transistors triggered by spikes (50 ms) with amplitudes 0.01, 0.05, 0.1, 0.5, 1, 1.5, 2, 2.25, 2.5, 2.75, 3, or 3.25 V.**

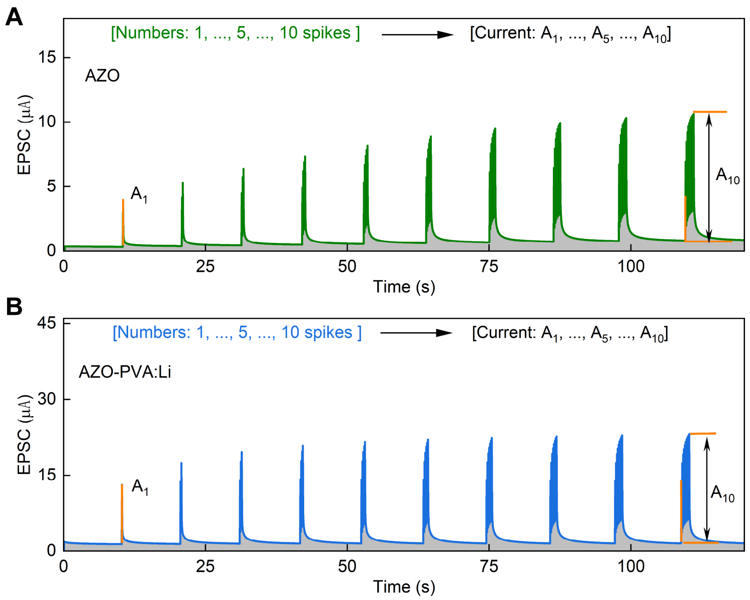


**Fig. S16. EPSC of AZO nanofibers-exploiting synaptic transistors triggered by consecutive spikes (2.5 V, 50 ms) with spike numbers 1, 2, 3, 4, 5, 6, 7, 8, 9, or 10.** (**A**) Without a modified layer. (**B**) With a modified layer.

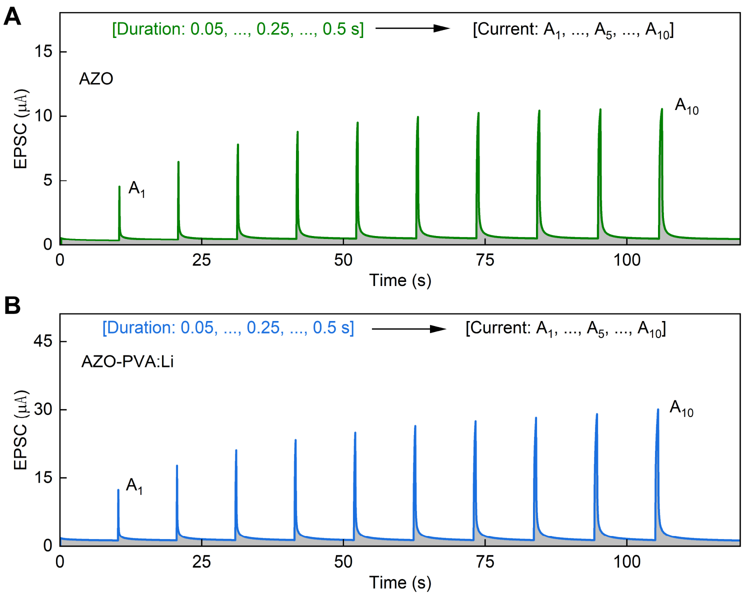


**Fig. S17. EPSC of AZO nanofibers-exploiting synaptic transistors triggered by spikes (2.5 V) with spike durations 0.05, 0.1, 0.15, 0.2, 0.25, 0.3, 0.35, 0.4, 0.45, or 0.5 s.** (**A**) Without a modified layer. (**B**) With a modified layer.

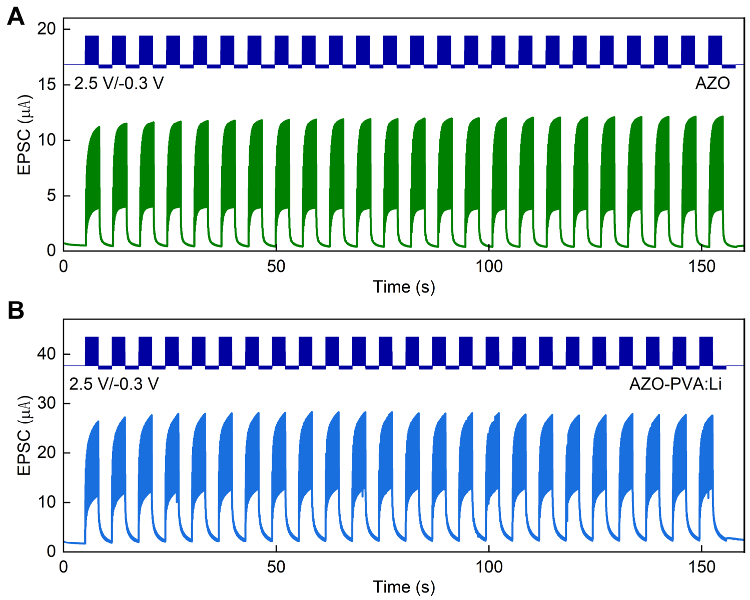


**Fig. S18. Twenty-four successive cycles of potentiation (30 spikes of 2.5 V) and depression (30 spikes of -0.3 V) for the AZO nanofibers-exploiting synaptic transistors.** (**A**) Without a modified layer. (**B**) With a modified layer.

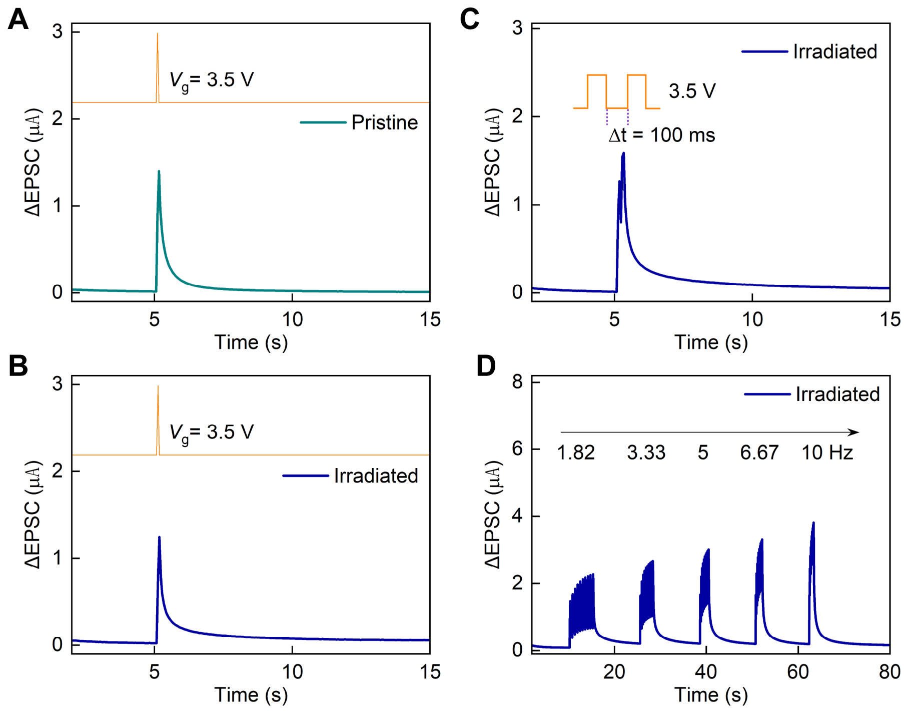


**Fig. S19. Performance of AZO nanofibers-exploiting synaptic transistors before and after proton irradiation of the AZO nanofibers at $V_d$ = 1 V.** (**A**) EPSC triggered by a single spike (3.5 V, 50 ms) before proton irradiation. (**B**) EPSC triggered by a single spike (3.5 V, 50 ms) after proton irradiation. (**C**) EPSC triggered by a pair of spikes (3.5 V, 50 ms) after proton irradiation. (**D**) EPSC triggered by ten consecutive spikes (3.5 V, 50 ms) with various spike frequencies (1.82, 3.33, 5, 6.67, and 10 Hz) after proton irradiation.

Note: The AZO nanofibers-exploiting synaptic transistors were fabricated on glass substrates, with patterned ITO films serving as the source and drain electrodes. The fabrication process for the AZO nanofibers and ion gels follows the procedures that were used on $Si/SiO_2$ substrates. The proton irradiation process for the AZO nanofibers is detailed in Supplementary Method 2, where 40 keV proton beams with a flux of $5 \times 10^{11}$ cm$^2$/s and a dose of $5 \times 10^{15}$ ions/cm$^2$ were used.

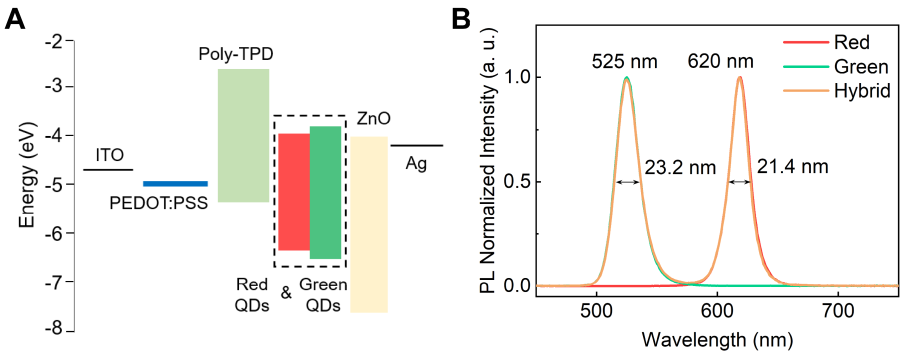


**Fig. S20. Energy band diagram of the hybrid QLED and PL spectra of different QDs.** (**A**) Energy band diagram. (**B**) PL spectra.

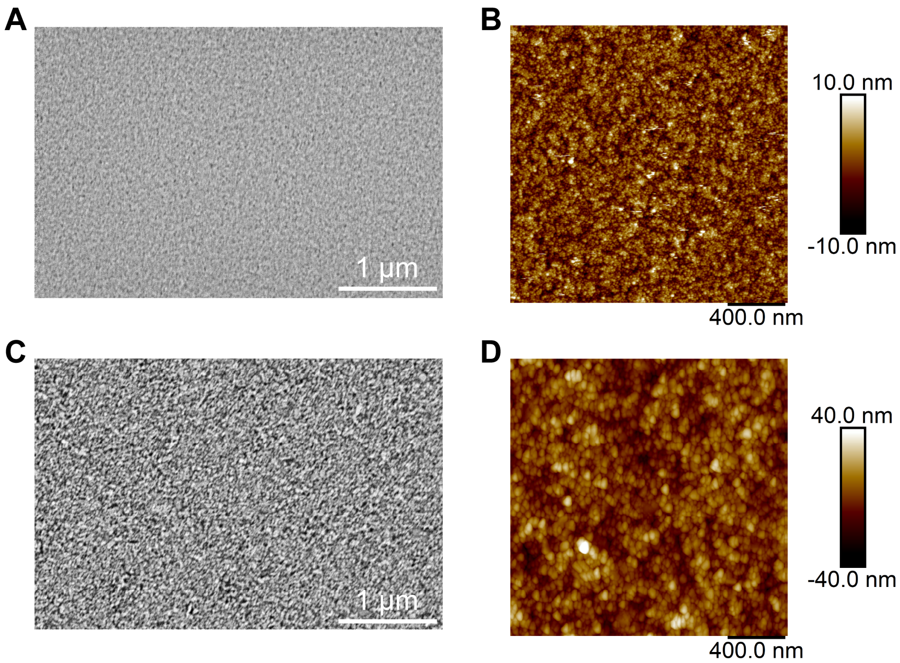


**Fig. S21. SEM and AFM images of the hybrid QDs layer and the ZnO nanoparticles layer in QLEDs.** (**A**), (**B**) Hybrid QDs layer. (**C**), (**D**) ZnO nanoparticles layer.

Note: The AFM images indicate that the average roughness (Ra) values for the hybrid QDs layer and the ZnO nanoparticles layer are 2.05 and 6.80 nm, respectively.

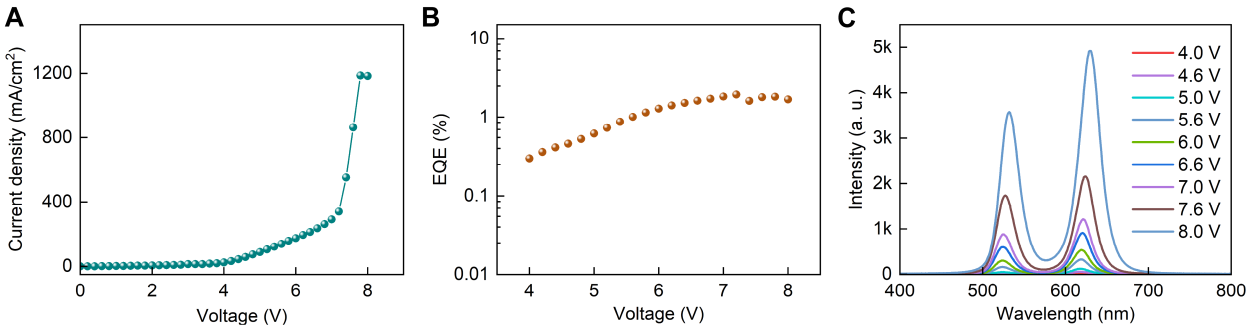


**Fig. S22. Current density–voltage (*C-V*) characteristics, external quantum efficiency (EQE), and EL spectra of the QLED.** (**A**) *C-V* characteristics. (**B**) EQE. (**C**) EL spectra under 4.0, 4.6, 5.0, 5.6, 6.0, 6.6, 7.0, 7.6, or 8 V.

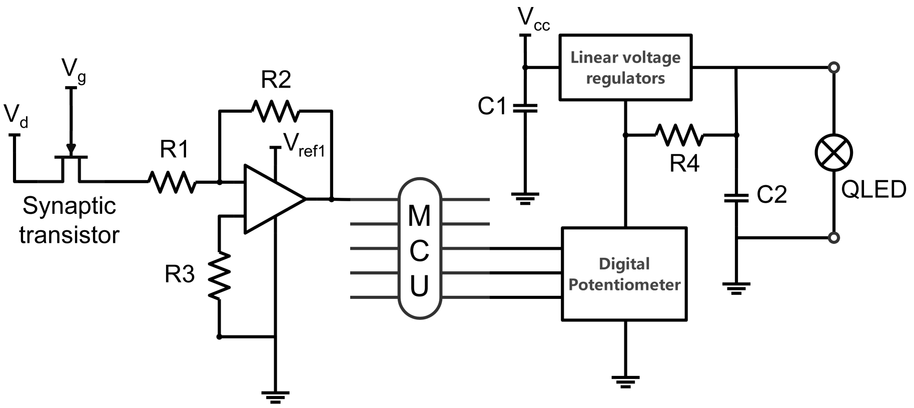


**Fig. S23. Schematic diagram of the integrated circuit for neuromorphic chromaticity encoding.**

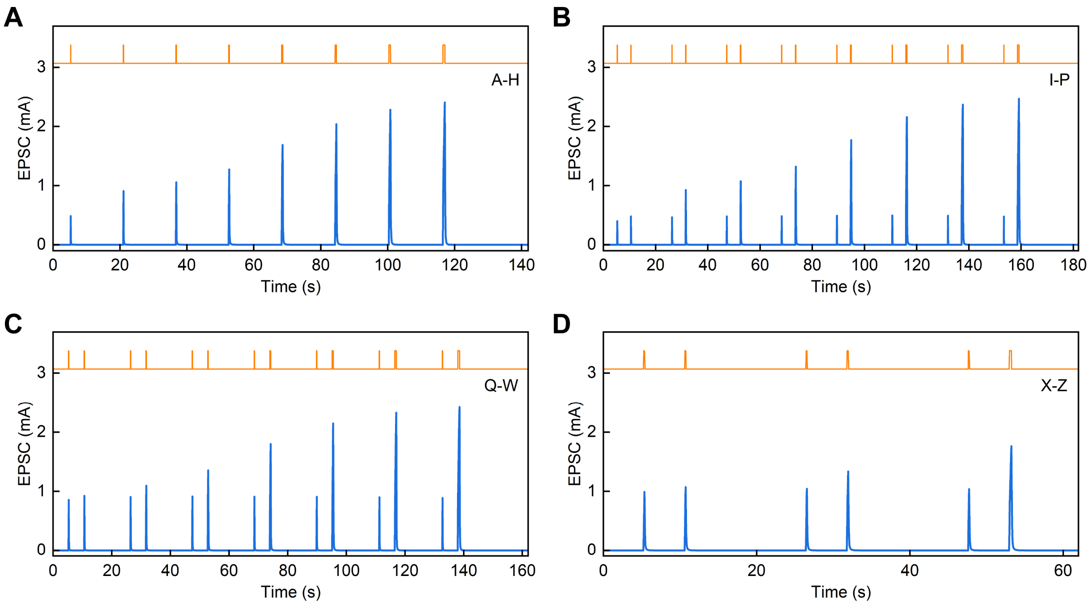


**Fig. S24. EPSCs corresponding to 26 English alphabet letters in neuromorphic chromaticity encoding.** (**A**) A-H. (**B**) I-P. (**C**) Q-W. (**D**) X-Z.

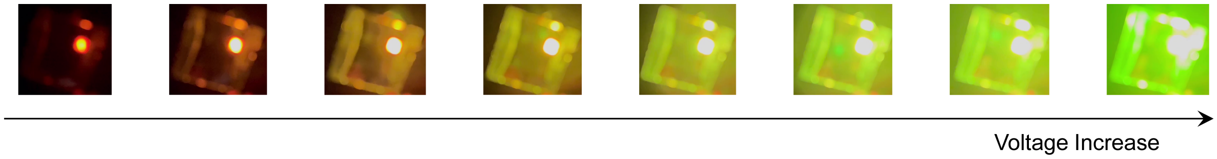


**Fig. S25. Photographs of the QLEDs driven by voltages of 4, 4.4, 5, 5.6, 6, 6.6, 7, and 8 V in neuromorphic chromaticity encoding.**

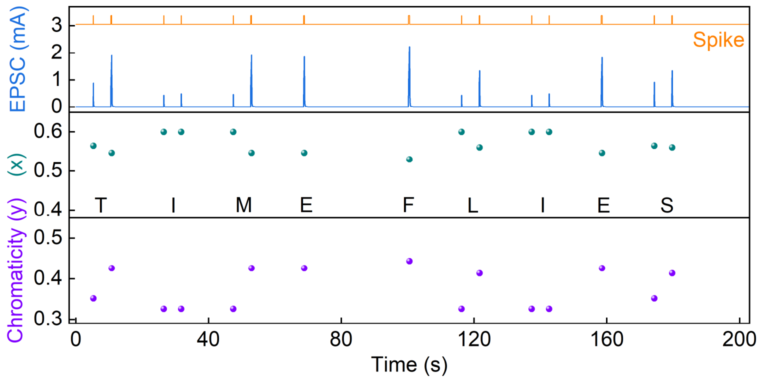


**Fig. S26. Presynaptic spikes, EPSC, and chromaticity corresponding to the phrase "Time flies" in neuromorphic chromaticity encoding.**

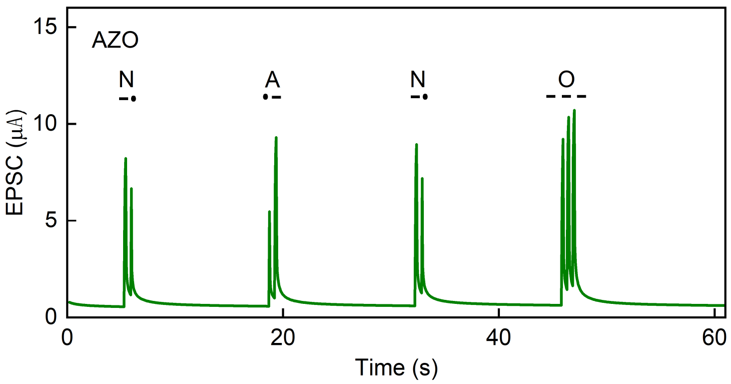


**Fig. S27. Morse code "NANO" generated by the AZO nanofibers-exploiting synaptic transistor without a modified layer.**

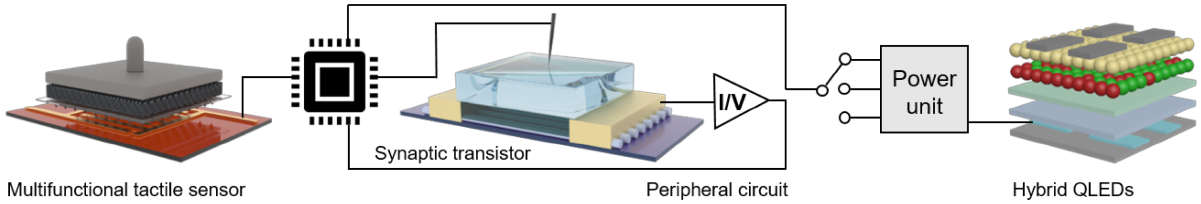


**Fig. S28. Schematic diagram of the configuration for the artificial neural circuit.**

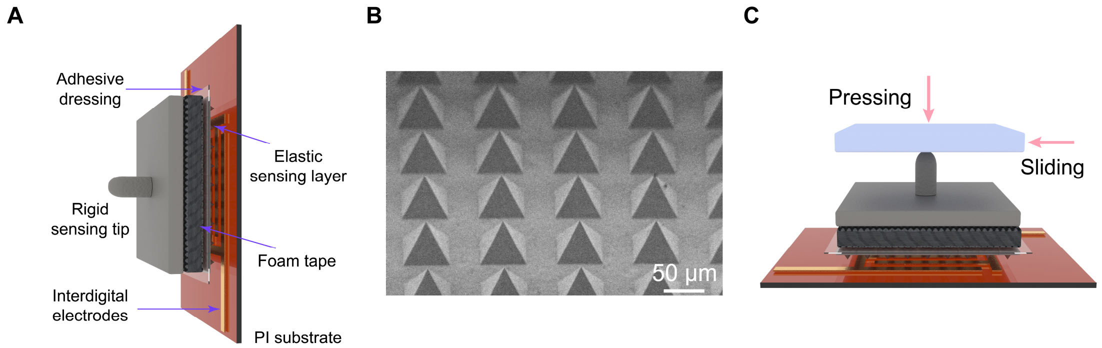


**Fig. S29. Structures and working modes of the multifunctional tactile sensor.** (**A**) Schematic of the multifunctional tactile sensor. (**B**) SEM image of the elastic sensing layer. (**C**) Schematic of the horizontal sliding and vertical pressing modes for the multifunctional tactile sensor.

Note: The multifunctional tactile sensor comprises a polyimide (PI) substrate, interdigital electrodes, an elastic sensing layer, an adhesive dressing, a foam tape, and a rigid sensing tip.

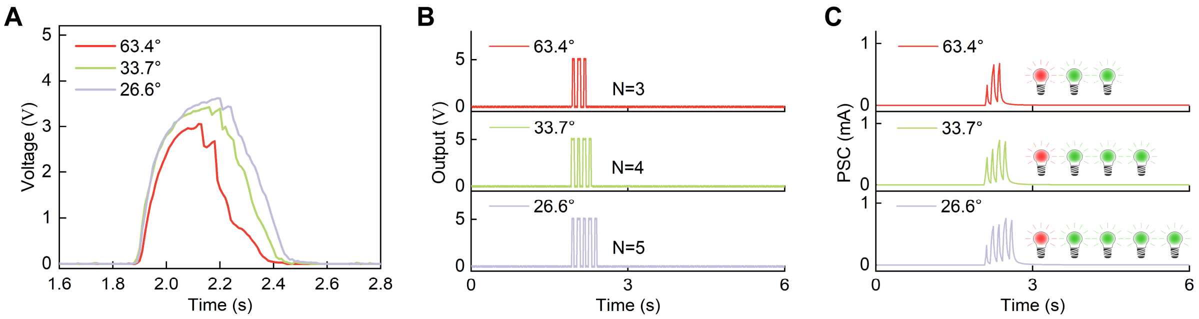


**Fig. S30. Response of the artificial neural circuit when detected stripes with different angles**. (**A**) Response of the multifunctional tactile sensor. (**B**) Output of the neuromorphic coding unit. (**C**) PSC of the AFST and corresponding color of light pulses.

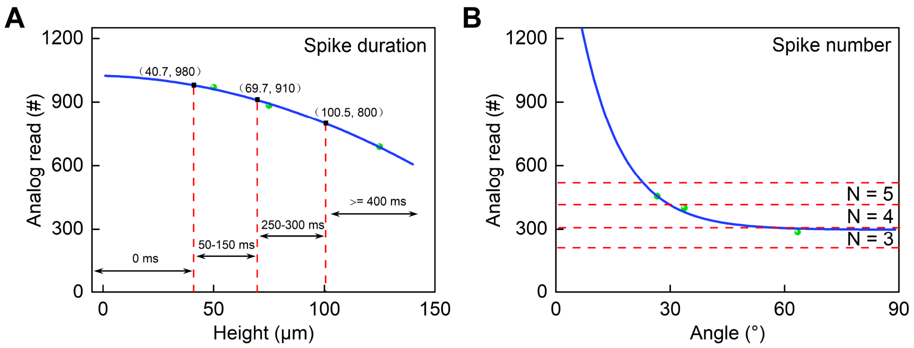


**Fig. S31. Presynaptic spikes output from the neuromorphic coding unit when the multifunctional tactile sensor detects stripes with different heights and angles.** (**A**) Stripes with different heights. (**B**) Stripes with different angles.

Note: The "Analog read" was obtained by Arduino, which corresponds to the response of the multifunctional tactile sensor. The "Analog read" value can convert to "voltage" via the equation: "Analog read" value/1024×5.

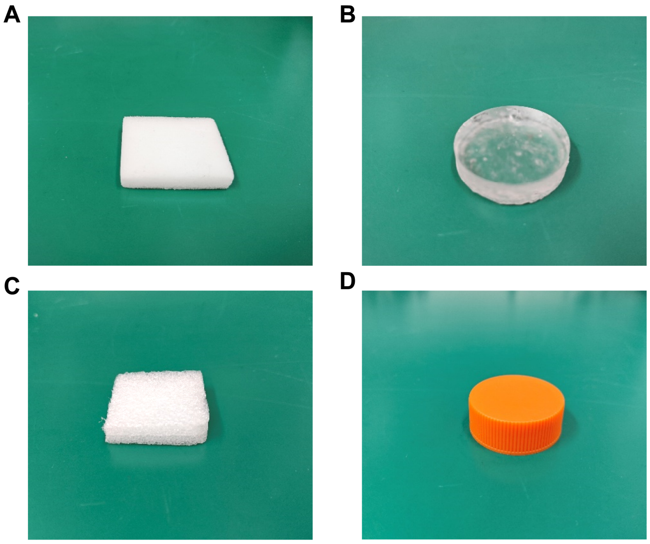


**Fig. S32. Digital images of the materials with different hardness detected by the multifunctional tactile sensor.** (**A**) Sponge. (**B**) Silicone elastomer. (**C**) Fiber foam. (**D**) Plastic.

Note: The hardness of the above materials are as follows:
Sponge < Silicone elastomer < Fiber foam < Plastic.

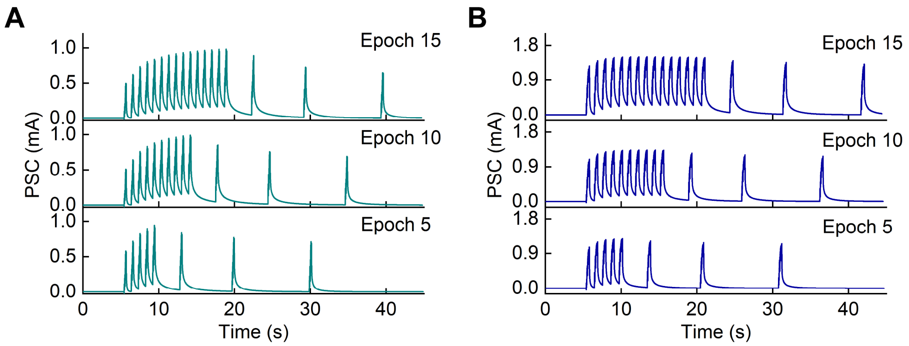


**Fig. S33. PSC of the AFST after different training epochs (5, 10, and 15) when detected stripes with heights of 75 and 125 μm.** (**A**) 75 μm. (**B**) 125 μm.

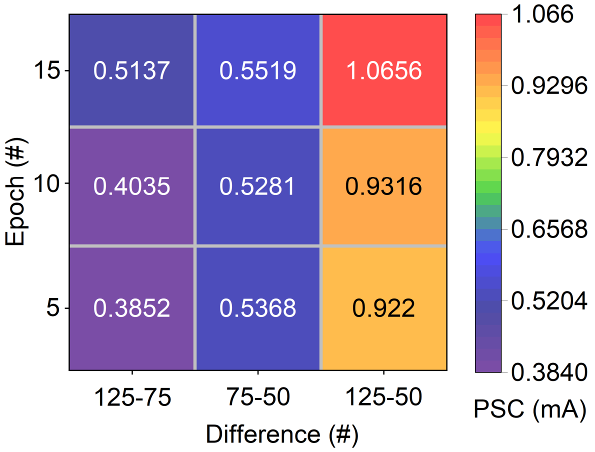


**Fig. S34. Discriminability of different stripes after training epochs of 5, 10, and 15.**

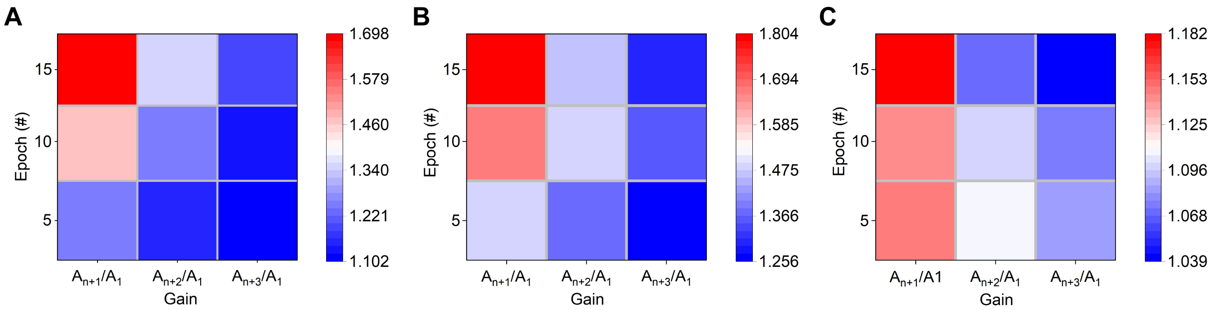


**Fig. S35. PSC gain of the AFST after different training epochs (5, 10, and 15) when detected stripes with heights of 50, 75, and 125 µm.** (**A**) 50 µm. (**B**) 75 µm. (**C**) 125 µm.

Note: PSC gain defined as $A_{n+1}/A_1$, $A_{n+2}/A_1$, and $A_{n+3}/A_1$. Here, $A_1$ is the PSC peak triggered by the first presynaptic spike, n is the training epoch.

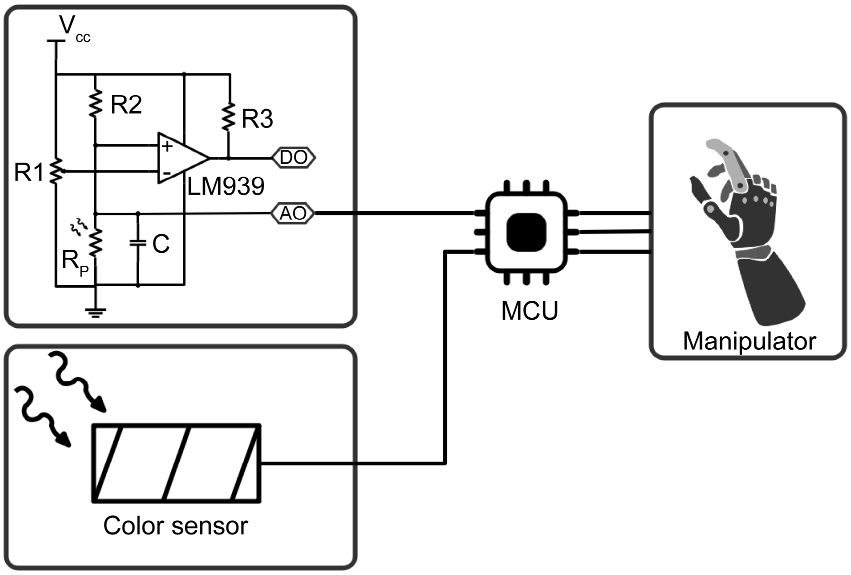


**Fig. S36. Diagram of the receiver used to control a manipulator.** Light signals are collected by the photosensitive resistor (up) and color sensor (down) to control the state of the manipulator.

Note: Commercially-available photosensitive resistor and color sensor were used to ensure the operational stability of the circuit.

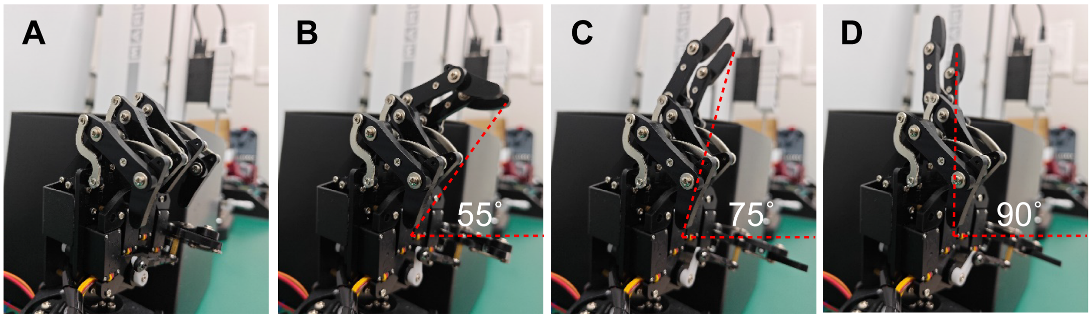


**Fig. S37. Digital images of the manipulator when the multifunctional tactile sensor detects stripes with different heights.** (**A**) Initial state**.** (**B**) Stripes of 50 μm. (**C**) Stripes of 75 μm. (**D**) Stripes of 125 μm.

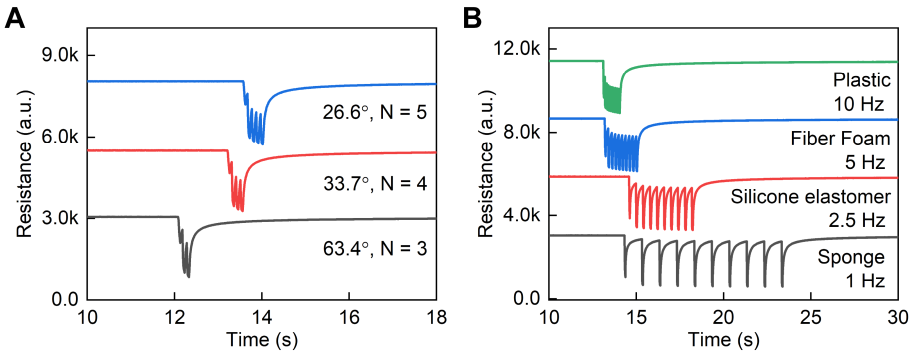


**Fig. S38. Light signals emitting from QLEDs collected by the photosensitive resistor when the multifunctional tactile sensor detects stripes with different angles and materials with different hardness.** (**A**) Stripes with different angles. (**B**) Materials with different hardness.

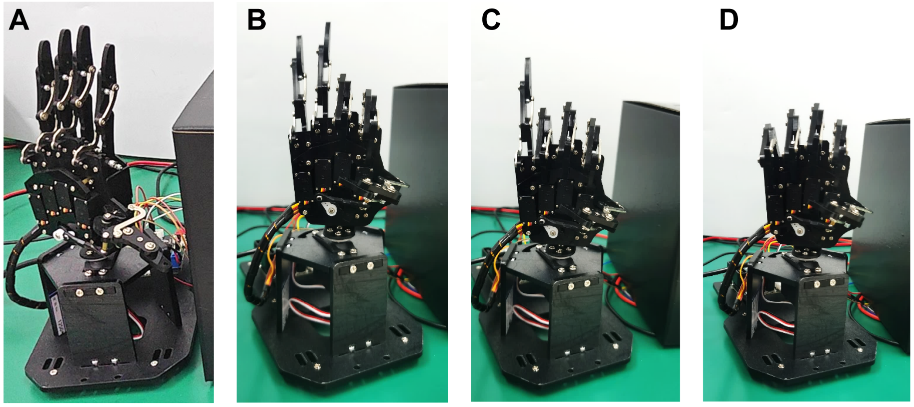


**Fig. S39. Digital images of the manipulator when the multifunctional tactile sensor detects stripes with different angles.** (**A**) Initial state. (**B**) Stripes of 63.4˚. (**C**) Stripes of 33.7˚. (**D**) Stripes of 26.6˚.

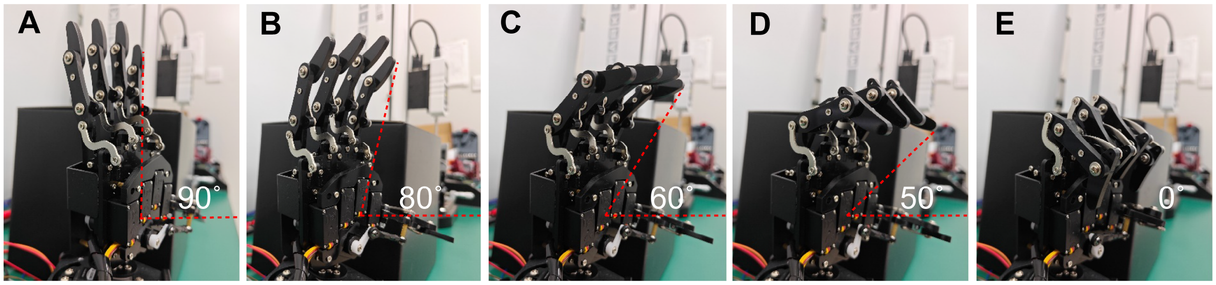


**Fig. S40. Digital images of the manipulator when the multifunctional tactile sensor detects materials with different hardness.** (**A**) Initial state. (**B**) Sponge. (**C**) Silicone elastomer. (**D**) Fiber foam. (**E**) Plastic.

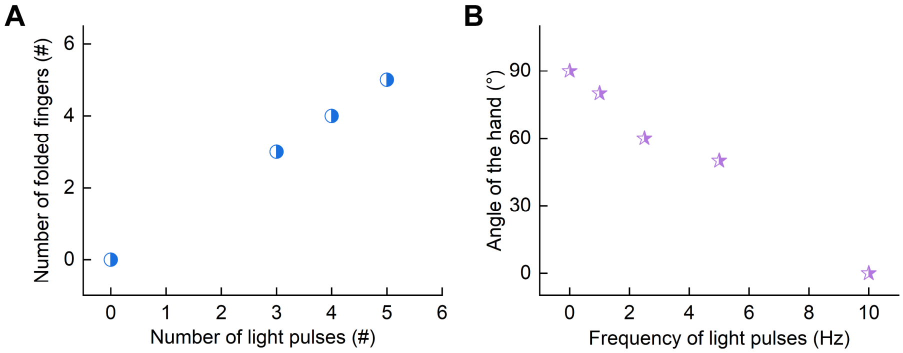


**Fig. S41. Quantity and frequency of light pulses emitted from the hybrid QLED, and the corresponding states of the manipulator.** (**A**) When detected stripes with different angles. (**B**) When detected materials with different hardness.

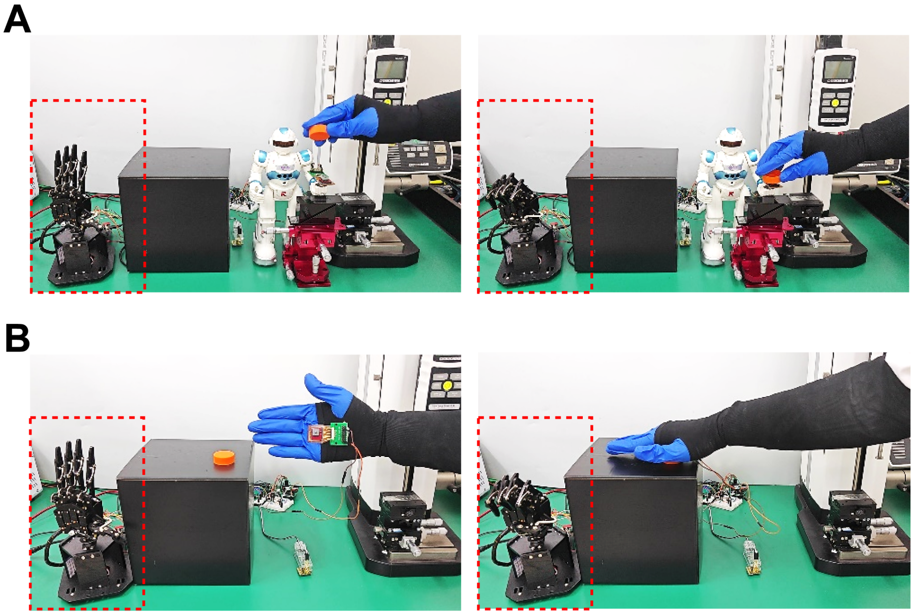

**Fig. S42. Digital images of the system equipped on a humanoid robot and the system for human-machine interaction.** (**A**) The system equipped on a humanoid robot. (**B**) The system for human-machine interaction.

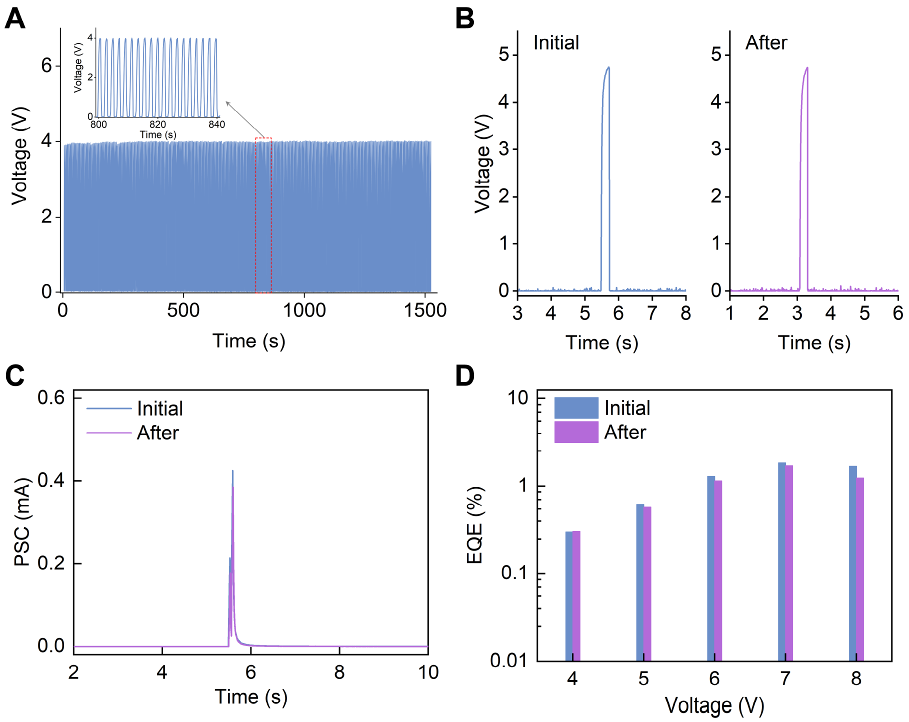


**Fig. S43. Operation of the key components within the NCCL.** (**A**) Cyclic stability of the multifunctional tactile sensor (~ 750 cycles). Stability of (**B**) multifunctional tactile sensor, (**C**) synaptic transistor, and (**D**) QLED, after being placed in an environment without water and oxygen for three months.

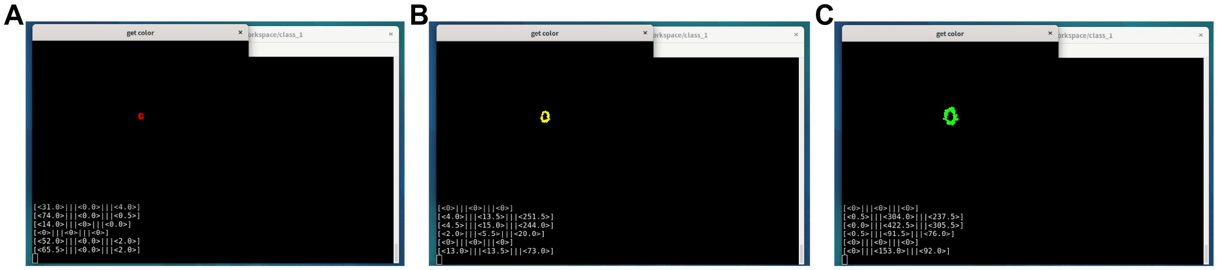


**Fig. S44. Emission areas of the QLED detected using the drone.** (**A**) Red light. (**B**) Orange light. (**C**) Green light.

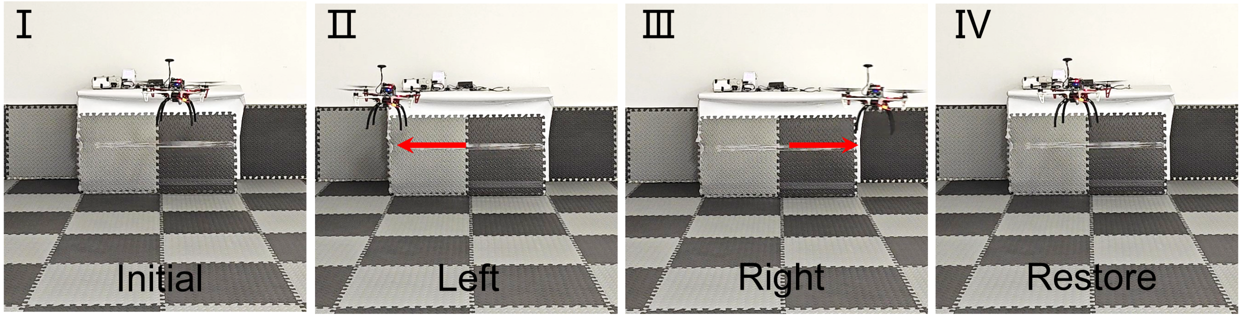


**Fig. S45. Digital images of flight maneuvers for the drone in response to orange light pulses.**

**Supplementary Tables**

**Table S1. Comparison of the irradiation tolerance to $H^+$ in various semiconductor materials**

| Material | Methods | Morphology | Irradiation duration (s) | Irradiation dose (ions/cm$^2$) |
|---|---|---|---|---|
| $Ga_2O_3$ (*56*) | / | Particles | 100 | $5\times10^{15}$ |
| $TiO_2$ (*56*) | / | Particles | 100 | $5\times10^{15}$ |
| ZnO (*57*) | Hydrothermal method | Particles | $1\times10^{4}$ | $5\times10^{15}$ |
| ZnO (*58*) | Thermal decomposition | Disordered nanorods | / | $5\times10^{15}$ |
| ZnO (*59*) | VPT | Single nanowire | $6\times10^{3}$ | $1\times10^{12}$ |
| ZnO (*60*) | VTD | Disordered nanowires | / | $5\times10^{15}$ |
| GaAs (*61*) | MSVLS | Disordered nanowires | / | $3\times10^{13}$ |
| InP (*61*) | MOVPE | Disordered nanowires | / | $5\times10^{13}$ |
| $MoS_2$ (*62*) | Mechanical exfoliation | 2D flakes | $2\times10^{3}$ | $1\times10^{14}$ |
| Graphene (*63*) | CVD | 2D flakes | / | $2\times10^{14}$ |
| $\alpha$-$MoO_3$ (*64*) | PVD | 2D flakes | / | $1\times10^{16}$ |
| 1T-$TaS_2$ (*65*) | CVT | Quasi-2D film | $2\times10^{3}$ | $1\times10^{14}$ |
| AZO (*66*) | Sputtering | Film | 100 | $1\times10^{14}$ |
| InP (*67*) | MBE | Film | $\sim 2.7\times10^{4}$ | $1\times10^{14}$ |

| | | | | |
|---|---|---|---|---|
| InAs (*68*) | MBE | Film | / | $7\times10^{14}$ |
| AlGaN/GaN (*69*) | MOCVD | Film | / | $6\times10^{14}$ |
| SnO (*70*) | Magnetron sputtering | Film | / | $1\times10^{14}$ |
| IZO (*27*) | Spin-coating | Film | 102 | $1\times10^{13}$ |
| ZTO (*17*) | Spin-coating | Film | 1018 | $1\times10^{15}$ |
| IGZO (*17*) | Spin-coating | Film | 1018 | $1\times10^{15}$ |
| FTO (*71*) | / | Film | / | $1\times10^{16}$ |
| IGTO (*72*) | Magnetron sputtering | Film | 102 | $1\times10^{13}$ |
| ITZO (*73*) | Spin-coating | Film | / | $1\times10^{15}$ |
| **AZO (This work)** | **Electrohydrod-ynamic printing** | **Highly aligned nanofibers** | $\mathbf{1\times10^{4}}$ | $\mathbf{5\times10^{15}}$ |

VPT: Vapor phase transport. VTD: Vapor transport deposition. MSVLS: Metal-seeded vapor-liquid-solid growth. MOVPE: Metalorganic vapor phase epitaxy. CVD: Chemical vapor deposition. PVD: Physical vapor deposition. CVT: Chemical vapor transport. MBE: Molecular beam epitaxy. MOCVD: Metal organic chemical vapor deposition.

**Table S2**. **Performance of synaptic transistors without and with a modified layer**

| Device | EPSC peak (2.5 V, 50 ms) | $V_{th}$ (V) | Sensitivity (mV) | Energy consumption (fJ/event) |
|---|---|---|---|---|
| AFST-wo | 4.19 μA | 2.28 | 1 | 1.33 |
| AFST-w | 10.38 μA | -0.14 | 1 | 4.55 |

**Table S3**. **PL properties of different QDs**

| QDs | PL peak (nm) | FWHM (nm) | TRPL decay (ns) |
|---|---|---|---|
| Red | 620 | 21.4 | 13.68 |
| Green | 525 | 23.2 | 9.90 |
| Hybrid | 620, 525 | 21.4, 23.2 | 7.00 |

FWHM: full widths at half maximum.

**Table S4**. **Response of the synaptic transistor, QLED, and manipulator when detected stripes with different heights**

| Height (μm) | Spike duration (ms) | PSC (mA) | Color of the light pulses | Angle of fingers (°) |
|---|---|---|---|---|
| 50 | 100 | 0.43 | Red | 55 |
| 75 | 250 | 0.67 | Orange | 75 |
| 125 | 400 | 0.81 | Green | 90 |

**Table S5**. **Response of the synaptic transistor, QLED, and manipulator when detected stripes with different angles**

| Angle (°) | Spike number | PSC (mA) | Number of the light pulses | Number of folded fingers |
|---|---|---|---|---|
| 63.4 | 3 | 0.68 | 3 | 3 |
| 33.7 | 4 | 0.71 | 4 | 4 |
| 26.6 | 5 | 0.72 | 5 | 5 |

**Table S6. Response of the synaptic transistor, QLED, and manipulator when detected materials with different hardness**

| Material | Spike frequency (Hz) | PSC (mA) | Frequency of the light pulses (Hz) | Angle of the hand (°) |
|---|---|---|---|---|
| Sponge | 1 | 0.50 | 1 | 80 |
| Silicone elastomer | 2.5 | 0.55 | 2.5 | 60 |
| Fiber Foam | 5 | 0.61 | 5 | 50 |
| Plastic | 10 | 0.68 | 10 | 0 |

**Supplementary Movies**

**Movie S1. Display of Morse Code and Logic Operation Using the Artificial Efferent Nerve**

**Movie S2. Conversion of Tactile Sensations into Optical Expression Using the Artificial Neural Circuit**

**Movie S3. Manipulator Control via Neuromorphic Chromaticity Communication Loop**

**Movie S4. Drone Flight Control via Neuromorphic Chromaticity Communication Loop**

## REFERENCES AND NOTES

1. P. Arm, G. Waibel, J. Preisig T. Tuna, R. Zhou V. Bickel, G. Ligeza T. Miki, F. Kehl, H. Kolvenbach, M. Hutter, Scientific exploration of challenging planetary analog environments with a team of legged robots. *Sci. Robot.* **8**, eade9548 (2023).

2. T. Reichhardt, A job for the droids? *Nature* **428**, 888–890 (2004).

3. T. Ghidini, Materials for space exploration and settlement. *Nat. Mater.* **17**, 846–850 (2018).

4. S. Chien, K. L. Wagstaff, Robotic space exploration agents. *Sci. Robot.* **2**, eaan4831 (2017).

5. X. Fang, W. Feng, Y. Chen, N. Ge, G. Zheng, Control-oriented deep space communications for unmanned space exploration. *IEEE Trans. Wirel. Commun.* **23**, 14466–14481 (2024).

6. G. Xu, Z. Song, Effects of solar scintillation on deep space communications: Challenges and prediction techniques. *IEEE Wirel. Commun.* **26**, 10–16 (2019).

7. Z. Feng, Z. Wei, X. Chen, H. Yang, Q. Zhang, P. Zhang, Joint communication, sensing, and computation enabled 6G intelligent machine system. *IEEE Network* **35**, 34–42 (2021).

8. Y. Gao, Chien S. Review on space robotics: Toward top-level science through space exploration. *Sci. Robot.* **2**, eaan5074 (2017).

9. S. Zhou, Y. Li, Q. Wang, Z. Lyu, Integrated actuation and sensing: Toward intelligent soft robots. *Cyborg Bionic Syst.* **5**, 0105 (2024).

10. S. Reiter, P. Hülsdunk, T. Woo, M. A. Lauterbach, J. S. Eberle, L. A. Akay, A. Longo, J. Meier-Credo, F. Kretschmer, J. D. Langer, M. Kaschube, G. Laurent, Elucidating the control and development of skin patterning in cuttlefish. *Nature* **562**, 361–366 (2018).

11. D. Scheel, P. Godfrey-Smith, M. Lawrence, Signal use by octopuses in agonistic interactions. *Curr. Biol.* **26**, 377–382 (2016).

12. B. P. Burford, B. H. Robison, Bioluminescent backlighting illuminates the complex visual signals of a social squid in the deep sea. *Proc. Natl. Acad. Sci. U.S.A.* **117**, 8524–8531 (2020).

13. J. B. Messenger Cephalopod chromatophores: Neurobiology and natural history. *Biol. Rev.* **76**, 473–528 (2007).

14. J. V. Logan, P. T. Webster, K. B. Woller, C. P. Morath, M. P. Short, Understanding the fundamental driver of semiconductor radiation tolerance with experiment and theory. *Phys. Rev. Mater.* **6**, 084601 (2022.

15. R. L. Pease, A. H. Johnston, J. L. Azarewicz, Radiation testing of semiconductor devices for space electronics. *Proc. IEEE* **76**, 1510–1526 (1988).

16. A. Azarov, J. G. Fernández, J. Zhao, F. Djurabekova, H. He, R. He, Prytz Ø. L. Vines, U. Bektas, P. Chekhonin, N. Klingner, G. Hlawacek, A. Kuznetsov, Universal radiation tolerant semiconductor. *Nat. Commun.* **14**, 4855 (2023).

17. B. Park, D. Ho, G. Kwon, D. Kim S. Y. Seo, C. Kim, M. G. Kim, Solution-processed rad-hard amorphous metal-oxide thin-film transistors. *Adv. Funct. Mater.* **28** 1802717 (2018).

18. R. Rasmidi, M. Duinong, F. P. Chee, Radiation damage effects on zinc oxide (ZnO) based semiconductor devices—A review. *Radiat. Phys. Chem.* **184**, 109455 (2021).

19. S. Qu, L. Sun, S. Zhang, J. Liu, Y. Li, J. Liu, W. Xu, An artificially-intelligent cornea with tactile sensation enables sensory expansion and interaction. *Nat. Commun.* **14**, 7181 (2023).

20. G. Kresse, J. Hafner, Ab initio molecular dynamics for open-shell transition metals. *Phys. Rev. B Condens. Matter* **48**, 13115–13118 (1993).

21. G. Kresse, J. Furthmüller, Efficiency of ab-initio total energy calculations for metals and semiconductors using a plane-wave basis set. *Comput. Mater. Sci.* **6**, 15–50 (1996).

22. W. Wang, Z. Wang, Y. Hu, Y. Liu, S. Chen, A potential-driven switch of activity promotion mode for the oxygen evolution reaction at $Co_3O_4/NiO_xH_y$ interface. *eScience* **2**, 438–444 (2022).

23. D. Dai, P. Wang, X. Bao, Y. Xu, Z. Wang, Y. Guo, Z. Wang, Z. Zheng, Y. Liu, H. Cheng, B. Huang, g-$C_3N_4$/ITO/Co-$BiVO_4$ Z-scheme composite for solar overall water splitting. *Chem. Eng. J.* **433**, 134476 (2022).

24. R. Raciti, R. Bahariqushchi, C. Summonte, A. Aydinli, A. Terrasi, S. Mirabella, Optical bandgap of semiconductor nanostructures: Methods for experimental data analysis. *J. Appl. Phys.* **121**, 234304 (2017).

25. K. Nomura, H. Ohta, A. Takagi, T. Kamiya, M. Hirano, H. Hosono, Room-temperature fabrication of transparent flexible thin-film transistors using amorphous oxide semiconductors. *Nature* **432**, 488–492 (2004).

26. Y. Fang, C. Zhao, I. Z. Mitrovic, C. Zhao, High-performance and radiation-hardened solution-processed ZrLaO gate dielectrics for large-area applications. *ACS Appl. Mater. Interfaces* **13**, 50101–50110 (2021).

27. Y. Kim, M.-G. Kim, C. Kim, Enhancing radiation-resistance of amorphous indium–zinc-oxide thin-film transistors by group IV transition element doping. *J. Mater. Chem. C* **11**, 10324–10332 (2023).

28. D. A. Bauman, A. I. Borodkin, A. A. Petrenko, D. I. Panov, A. V. Kremleva, V. A. Spiridonov, D. A. Zakgeim, M. V. Silnikov, M. A. Odnoblyudov, A. E. Romanov, V. E. Bougrov, On improving the radiation resistance of gallium oxide for space applications. *Acta Astronaut.* **180**, 125–129 (2021).

29. H. H.-C. Lai, T. Basheer V. L. Kuznetsov, R. G. Egdell, R. M. J. Jacobs M. Pepper, P. P. Edwards, Dopant-induced bandgap shift in Al-doped ZnO thin films prepared by spray pyrolysis. *J. Appl. Phys.* **112**, 083708 (2012).

30. F. Dubas, R. T. Hanlon, G. P. Ferguson, H. M. Pinsker, Localization and stimulation of chromatophore motoneurones in the brain of the squid, lolliguncula brevis. *J. Exp. Biol.* **121**, 1–25 (1986).

31. C. M. Reed, The ultrastructure and innervation of muscles controlling chromatophore expansion in the squid, *Loligo vulgaris*. *Cell Tissue Res.* **282**, 503–512 (1995).

32. E. Florey, F. Dubas, R. T. Hanlon, Evidence for l-glutamate as a transmitter substance of motoneurons innervating squid chromatophore muscles. *Comp. Biochem. Phys. C* **82**, 259–268 (1985).

33. S. Kunigal Vijaya Shankar, Y. Claveau, T. Rasoanarivo, C. Ewels, J. Le Bideau, Impact of Li, Na and Zn metal cation concentration in EMIM–TFSI ionic liquids on ion clustering, structure and dynamics. *Phys. Chem. Chem. Phys.* **26**, 7049–7059 (2024).

34. A. Subramanian, B. George, S. R. Bobbara, I. Valitova, I. Ruggeri, F. Borghi, A. Podestà, P. Milani, F. Soavi, C. Santato, F. Cicoira, Ion-gated transistors based on porous and compact $TiO_2$ films: Effect of Li ions in the gating medium. *AIP Adv.* **10**, 065314 (2020).

35. J. Wang, Z. Zhao, S. Song, Q. Ma, R. Liu, High performance poly(vinyl alcohol)-based Li-ion conducting gel polymer electrolyte films for electric double-layer capacitors. *Polymers* **10**, 1179 (2018).

36. R. He, A. Lv, X. Jiang, C. Cai, Y. Wang, W. Yue, L. Huang, X. B. Yin, L. Chi, Organic electrochemical transistor based on hydrophobic polymer tuned by ionic gels. *Angew. Chem.* **62**, e202304549 (2023).

37. L. F. Abbott, W. G. Regehr, Synaptic computation. *Nature* **431**, 796–803 (2004).

38. T. J. Lee K. R. Yun, S. K. Kim J. H. Kim, J. Jin, K. B. Sim, D. H. Lee G. W. Hwang, T. Y. Seong, Realization of an artificial visual nervous system using an integrated optoelectronic device array. *Adv. Mater.* **33**, e2105485 (2021).

39. H. Shen Q. Gao, Y. Zhang, Y. Lin, Q. Lin, Z. Li, L. Chen, Z. Zeng, X. Li, Y. Jia, S. Wang, Z. Du, L. S. Li, Z. Zhang, Visible quantum dot light-emitting diodes with simultaneous high brightness and efficiency. *Nat. Photon.* **13**, 192–197 (2019).

40. Y. Zhu, R. Xu, Y. Zhou, Z. Xu, Y. Liu, F. Tian, X. Zheng, F. Ma, R. Alsharafi, H. Hu, T. Guo, T. W. Kim, F. Li, Ultrahighly efficient white quantum dot light-emitting diodes operating at low voltage. *Adv. Opt. Mater.* **8**, 2001479 (2020).

41. T.-L. Shen, H.-W. Hu, W.-J. Lin, Y.-M. Liao, T.-P. Chen, Y.-K. Liao, Lin T.-Y., Y.-F. Chen, Coherent Förster resonance energy transfer: A new paradigm for electrically driven quantum dot random lasers. *Sci. Adv.* **6**, eaba1705 (2020).

42. J. Ren, T. Wu, B. Yang, X. Zhang, Simultaneously giant enhancement of Förster resonance energy transfer rate and efficiency based on plasmonic excitations. *Phys. Rev. B* **94**, 125416 (2016).

43. R. Yuste, R. Cossart, E. Yaksi, Neuronal ensembles: Building blocks of neural circuits. *Neuron* **112**, 875–892 (2024).

44. M. Rosas-Ballina, P. S. Olofsson, M. Ochani, S. I. Valdés-Ferrer, Y. A. Levine, C. Reardon, M. W. Tusche, V. A. Pavlov, U. Andersson, S. Chavan, T. W. Mak, K. J. Tracey, Acetylcholine-synthesizing T cells relay neural signals in a vagus nerve circuit. *Science* **334**, 98–101 (2011).

45. L. Luo, Architectures of neuronal circuits. *Science* **373**, 1103 (2021).

46. J. P. Perdew, K. Burke, M. Ernzerhof, Generalized gradient approximation made simple. *Phys. Rev. Lett.* **77**, 3865–3868 (1996).

47. P. E. Blochl, Projector augmented-wave method. *Phys. Rev. B* **50**, 17953–17979 (1994).

48. S. H. Wei, L. G. Ferreira, J. E. Bernard, A. Zunger, Electronic properties of random alloys: Special quasirandom structures. *Phys. Rev. B* **42**, 9622–9649 (1990).

49. A. Zunger, S. Wei, L. G. Ferreira, J. E. Bernard, Special quasirandom structures. *Phys. Rev. Lett.* **65**, 353–356 (1990).

50. V. Wang, N. Xu, J.-C. Liu, G. Tang, W.-T. Geng, VASPKIT: A user-friendly interface facilitating high-throughput computing and analysis using VASP code. *Comput. Phys. Commun.* **267**, 108033 (2021).

51. J. Stachurski, S. Tamariz, G. Callsen, R. Butté, N. Grandjean, Single photon emission and recombination dynamics in self-assembled GaN/AlN quantum dots. *Light Sci. Appl.* **11**, 114 (2022).

52. D. Bi, C. Yi, J. Luo, J.-D. Décoppet, F. Zhang, S. M. Zakeeruddin, X. Li, A. Hagfeldt, M. Grätzel, Polymer-templated nucleation and crystal growth of perovskite films for solar cells with efficiency greater than 21%. *Nat. Energy* **1**, 16142 (2016).

53. H. Zhang, Q. Su, S. Chen, Quantum-dot and organic hybrid tandem light-emitting diodes with multi-functionality of full-color-tunability and white-light-emission. *Nat. Commun.* **11**, 2826 (2020).

54. J. Gong, Y. Wei, Y. Wang, Z. Feng, J. Yu, L. Cheng, M. Chen, L. Li, Z. L. Wang, Q. Sun, Brain-inspired multimodal synaptic memory via mechano-photonic plasticized asymmetric ferroelectric heterostructure. *Adv. Funct. Mater.* **34**, 2408435 (2024).

55. W. Xu, S.-Y. Min, H. Hwang, T.-W. Lee, Organic core-sheath nanowire artificial synapses with femtojoule energy consumption. *Sci. Adv.* **2**, e1501326 (2016).

56. T. Lu, J. Lv, C. Wang, Hydrogenation process enhances radiation-stability of ZnO, $Ga_2O_3$ and $TiO_2$. *J. Alloys Compd.* **897**, 163135 (2022).

57. V. Neshchimenko, C. Li, M. Mikhailov, J. Lv, Optical radiation stability of ZnO hollow particles. *Nanoscale* **10**, 22335–22347 (2018).

58. J. Lv, C. Li, Y. Liu Oxygen-deficient defects facilitate $H^+$ radiation resistance in ZnO. *Phys. Status Solidi B* **254**, 1600411 (2016).

59. W.-K. Hong, G. Jo, J. I. Sohn, W. Park, M. Choe, G. Wang, Y. H. Kahng, M. E. Welland, T. Lee, Tuning of the electronic characteristics of ZnO nanowire field effect transistors by proton irradiation. *ACS Nano* **4**, 811–818 (2010).

60. C. F. Dee, I. Ahmad, L. Yan, X. Zhou, B. Y. Majlis, Amorphization of ZnO nanowires by proton beam irradiation. *Nano* **06**, 259–263 (2011).

61. F. Li, X. Xie, Q. Gao, L. Tan, Y. Zhou, Q. Yang, J. Ma, L. Fu, H. H. Tan, C. Jagadish, Enhancement of radiation tolerance in GaAs/AlGaAs core–Shell and InP nanowires. *Nanotechnology* **29**, 225703 (2018).

62. T.-Y. Kim, K. Cho, W. Park, J. Park, Y. Song, S. Hong, W.-K. Hong, T. Lee, Irradiation effects of high-energy proton beams on $MoS_2$ field effect transistors. *ACS Nano* **8**, 2774–2781 (2014).

63. S. Lee, J. Seo, J. Hong, S. H. Park, J.-H. Lee, B.-W. Min, T. Lee, Proton irradiation energy dependence of defect formation in graphene. *Appl. Surf. Sci.* **344**, 52–56 (2015).

64. R. Kumar, V. Mishra, T. Dixit, S. N. Sarangi, D. Samal, M. Miryala, P. K. Nayak, M. S. R. Rao, Investigating the effect of $H^+$-ion irradiation on layered $\alpha$-$MoO_3$ flakes by defect engineering. *Appl. Phys. Lett.* **123**, 151104 (2023).

65. A. K. Geremew, F. Kargar, E. X. Zhang, S. E. Zhao, E. Aytan, M. A. Bloodgood, T. T. Salguero, S. Rumyantsev, A. Fedoseyev, D. M. Fleetwood, A. A. Balandin, Proton-irradiation-immune electronics implemented with two-dimensional charge-density-wave devices. *Nanoscale* **11**, 8380–8386 (2019).

66. S. K. Sahoo, S. Mangal, D. K. Mishra, U. P. Singh, P. Kumar, 50 keV $H^+$ ion beam irradiation of Al doped ZnO thin films: Studies of radiation stability for device applications. *Surf. Interface Anal.* **49**, 1279–1286 (2017).

67. J.-L. Zhang, P. Ding, B. Mei S.-H. Meng, C. Zhang, L.-H. Ma, Z. Jin Y. Sun, H.-M. Zhang, Y.-H. Zhong, The effects and mechanisms of 2 MeV proton irradiation on InP-based high electron mobility transistors. *Appl. Phys. Lett.* **120**, 103501 (2022).

68. B. D. Weaver, J. B. Boos, N. A. Papanicolaou B. R. Bennett, D. Park, R. Bass, High radiation tolerance of InAs/AlSb high-electron-mobility transistors. *Appl. Phys. Lett.* **87** 173501 (2005).

69. J. D. Greenlee, P. Specht, T. J. Anderson, A. D. Koehler, B. D. Weaver, M. Luysberg, O. D. Dubon, F. J. Kub, T. R. Weatherford, K. D. Hobart, Degradation mechanisms of 2 MeV proton irradiated AlGaN/GaN HEMTs. *Appl. Phys. Lett.* **107**, 083504 (2015).

70. H.-Y. Jeong, S.-H. Kwon, H.-J. Joo, M.-G. Shin, H.-S. Jeong, D.-H. Kim, H.-I. Kwon, Radiation-tolerant p-type SnO thin-film transistors. *IEEE Electron. Device Lett.* **40**, 1124–1127 (2019).

71. B. Oryema, E. Jurua, I. G. Madiba, I. Ahmad, S. O. Aisida, F. I. Ezema, M. Maaza, Effects of 7 MeV proton irradiation on microstructural, morphological, optical, and electrical properties of fluorine-doped tin oxide thin films. *Surf. Interfaces* **28**, 101693 (2022).

72. M.-G. Shin, S.-H. Hwang, H.-S. Cha, H.-S. Jeong, D.-H. Kim, H.-I. Kwon, Effects of proton beam irradiation on the physical and chemical properties of IGTO thin films with different thicknesses for thin-film transistor applications. *Surf. Interfaces* **23**, 100990 (2021).

73. D. Ho, S. Choi, H. Kang, B. Park, M. N. Le, S. K. Park, M.-G. Kim, C. Kim, A. Facchetti, In situ radiation hardness study of amorphous Zn–In–Sn–O thin-film transistors with structural plasticity and defect tolerance. *ACS Appl. Mater. Interfaces* **15**, 33751–33762 (2023).
